# Cost-effectiveness of Low-complexity Screening Tests in Community-based Case-finding for Tuberculosis

**DOI:** 10.1093/cid/ciad501

**Published:** 2023-08-25

**Authors:** Lukas E Brümmer, Ryan R Thompson, Akash Malhotra, Sourya Shrestha, Emily A Kendall, Jason R Andrews, Patrick Phillips, Payam Nahid, Adithya Cattamanchi, Florian M Marx, Claudia M Denkinger, David W Dowdy

**Affiliations:** Division of Infectious Disease and Tropical Medicine, Center for Infectious Diseases, Heidelberg University Hospital, Heidelberg, Germany; German Center for Infection Research (DZIF), partner site Heidelberg, Heidelberg University Hospital, Heidelberg, Germany; Department of Epidemiology, Johns Hopkins Bloomberg School of Public Health, Baltimore, Maryland, USA; Department of Epidemiology, Johns Hopkins Bloomberg School of Public Health, Baltimore, Maryland, USA; Department of Epidemiology, Johns Hopkins Bloomberg School of Public Health, Baltimore, Maryland, USA; Department of Epidemiology, Johns Hopkins Bloomberg School of Public Health, Baltimore, Maryland, USA; Department of Epidemiology, Johns Hopkins Bloomberg School of Public Health, Baltimore, Maryland, USA; Center for Tuberculosis Research, Division of Infectious Diseases, Johns Hopkins University School of Medicine, Baltimore, Maryland, USA; Division of Infectious Diseases and Geographic Medicine, Department of Medicine, Stanford University, San Francisco, California, USA; Center for Tuberculosis, Division of Pulmonary and Critical Care Medicine, University of California, San Francisco, California, USA; Center for Tuberculosis, Division of Pulmonary and Critical Care Medicine, University of California, San Francisco, California, USA; Center for Tuberculosis, Division of Pulmonary and Critical Care Medicine, University of California, San Francisco, California, USA; Division of Pulmonary Diseases and Critical Care Medicine, University of California Irvine, Irvine, California, USA; Division of Infectious Disease and Tropical Medicine, Center for Infectious Diseases, Heidelberg University Hospital, Heidelberg, Germany; German Center for Infection Research (DZIF), partner site Heidelberg, Heidelberg University Hospital, Heidelberg, Germany; Division of Infectious Disease and Tropical Medicine, Center for Infectious Diseases, Heidelberg University Hospital, Heidelberg, Germany; German Center for Infection Research (DZIF), partner site Heidelberg, Heidelberg University Hospital, Heidelberg, Germany; Department of Epidemiology, Johns Hopkins Bloomberg School of Public Health, Baltimore, Maryland, USA

**Keywords:** tuberculosis, diagnostics, screening, cost-effectiveness, mathematical modelling

## Abstract

**Introduction:**

In high-burden settings, low-complexity screening tests for tuberculosis (TB) could expand the reach of community-based case-finding efforts. The potential costs and cost-effectiveness of approaches incorporating these tests are poorly understood.

**Methods:**

We developed a microsimulation model assessing 3 approaches to community-based case-finding in hypothetical populations (India-, South Africa-, The Philippines-, Uganda-, and Vietnam-like settings) with TB prevalence 4 times that of national estimates: (1) screening with a point-of-care C-reactive protein (CRP) test, (2) screening with a more sensitive “Hypothetical Screening test” (95% sensitive for Xpert Ultra-positive TB, 70% specificity; equipment/labor costs similar to Xpert Ultra, but using a $2 cartridge) followed by sputum Xpert Ultra if positive, or (3) testing all individuals with sputum Xpert Ultra. Costs are expressed in 2023 US dollars and include treatment costs.

**Results:**

Universal Xpert Ultra was estimated to cost a mean $4.0 million (95% uncertainty range: $3.5 to $4.6 million) and avert 3200 (2600 to 3900) TB-related disability-adjusted life years (DALYs) per 100 000 people screened ($670 [The Philippines] to $2000 [Vietnam] per DALY averted). CRP was projected to cost $550 (The Philippines) to $1500 (Vietnam) per DALY averted but with 44% fewer DALYs averted. The Hypothetical Screening test showed minimal benefit compared to universal Xpert Ultra, but if specificity were improved to 95% and per-test cost to $4.5 (all-inclusive), this strategy could cost $390 (The Philippines) to $940 (Vietnam) per DALY averted.

**Conclusions:**

Screening tests can meaningfully improve the cost-effectiveness of community-based case-finding for TB but only if they are sensitive, specific, and inexpensive.

## Teaser

Active case-finding might reduce the tuberculosis (TB) diagnostic gap. We estimate that screening tests could meaningfully improve these active case-finding efforts, but only if they are highly sensitive, specific (approximately 70%–95%), and available at low costs (approaching $4.5/test all-inclusive).

Approximately 4 million people develop tuberculosis (TB) each year who are never notified to public health authorities and/or never receive adequate treatment [1]. An estimated 36%–80% of people with prevalent TB do not have typical symptoms; thus, only strengthening routine testing of people presenting with such symptoms will leave a large proportion of TB undiagnosed [2]. Active case finding strategies, including community-based systematic screening, hold potential to improve TB diagnosis and linkage to care, but the optimal screening tools for different populations remain uncertain [3].

For molecular tests such as Xpert MTB/RIF Ultra (“Xpert Ultra”; Cepheid, Sunnyvale, California, United States) that are designed to diagnose TB in clinical settings, high cost hampers their use as the initial test in community-based case-finding efforts [4]. However, using low-complexity tests for screening the entire population of interest, followed by a more accurate, sputum-based molecular test to confirm diagnoses among those who screened positive, might make community-based case-finding more cost-effective [5]. Chest radiography could be a feasible screening tool but requires significant capital investment, moderate technical expertise and infrastructure, and radiation safety approvals, making it less feasible for many resource-limited settings [5]. Given that chest X-ray is unlikely to be a universally available screening tool, it is important to evaluate other tests that could be implemented with greater ease.

Several screening assays are in development that could be used for community-based case finding efforts, utilizing readily available clinical specimens such as capillary blood. These assays might use rapid point-of-care technology, enabling immediate decisions regarding confirmatory testing, or might require modest laboratory infrastructure to reach a decision regarding confirmatory testing. The point-of-care iChroma C-reactive protein test (CRP; Boditech, Chuncheon-si, Gang-won-do, South Korea) can be viewed as representative of the first use case, and the 3-gene Xpert MTB Host Response test (Cepheid, Sunnyvale, California, United States) of the second [6, 7]. In their current forms, point-of-care CRP lacks sufficient sensitivity to be used in most community-based screening, and Xpert MTB Host Response is too costly for this purpose. Despite not representing optimal screening tests in their current forms, point-of-care CRP and the Xpert MTB Host Response test may be considered as precursors to improved low-complexity TB screening assays that could be developed in the coming years.

To guide the development of these assays and their implementation for community-based case-finding, we analyzed the cost-effectiveness of 2 screening tests in high-burden settings: point-of-care CRP (in its current form) and a hypothetical screening test representing lower cost and higher accuracy relative to Xpert MTB Host Response. Our primary objectives were to evaluate the cost savings that could be achieved with these 2 assays, assess their cost-effectiveness relative to universal Xpert Ultra testing, and evaluate their ability to make community-based case-finding cost-effective (relative to no case-finding) in high-burden countries.

## METHODS

### Model Design and Parameters

We used a microsimulation Markov model to estimate the cost-effectiveness of different approaches for community-based TB case-finding. We assumed that individuals in target communities would follow a diagnostic and care cascade consisting of screening, followed by sputum Xpert Ultra testing to confirm the diagnosis of those who screened positive, and treatment initiation if the Xpert Ultra test was positive [3]. As people with Xpert Ultra-negative TB would not be detected using any algorithm, we benchmarked sensitivity against that of Xpert Ultra (ie, assessed sensitivity for Xpert Ultra-positive TB). We assumed 98.8% specificity for Xpert Ultra [5]. Four different screening scenarios were considered:

1) **No case-finding:** We projected costs and epidemiological effects with no community-based TB case-finding efforts.
2) **Point-of-care CRP screening:** CRP as the screening test, with a threshold of ≥5 mg/L for confirmatory testing (65% sensitivity for Xpert Ultra-positive TB and 84% specificity).
3) **Hypothetical Screening test:** A hypothetical screening test, assumed to represent different improvements on Xpert MTB/RIF Host Response:
  (a) Baseline scenario—95% sensitivity for Xpert Ultra-positive TB, 70% specificity, and use of the GeneXpert (Cepheid, Sunnyvale, California, United States) platform with a $2 cartridge cost, assuming non-cartridge (eg, equipment/labor) costs equivalent to those of Xpert (total per-test costs between $18.90 [India] and $34.85 [South Africa] [8–11]).
  (b) As in Scenario 3a but with costs similar to those of point-of-care CRP ($4.50 per test, all-inclusive).
  (c) As in Scenario 3b but with specificity also increased to 95%.
4) **Xpert Ultra for all:** Universal Xpert Ultra without a separate screening test. Considering only Xpert Ultra-positive TB, we model Xpert Ultra sensitivity as 100% and specificity as 98.8%.

The Hypothetical Screening test was motivated by evaluations of the Xpert MTB/RIF Host-Response within the Rapid Research in Diagnostics Development (R2D2) TB Network, a multi-country research consortium which evaluates novel diagnostic solutions for TB [12]. In R2D2, Xpert MTB/RIF Host-Response—when used in clinical rather than community-based case-finding settings—showed an accuracy profile close to the minimal requirements of the WHO target product profile for triage tests [13]. We reasoned that future assays might be able to achieve comparable levels of accuracy in the context of community-based screening.

We chose 5 countries participating in R2D2 as example settings: India, the Philippines, South Africa, Uganda, and Vietnam. For each country, each of the 4 screening strategies was simulated in a theoretical population of 100 000 individuals in a community setting, assuming no access to chest X-ray. We considered losses to follow-up (LTFU) prior to Xpert Ultra testing (eg, individuals unable/unwilling to expectorate a sufficient volume of sputum) and prior to treatment [14–20]. Considering that the factors (eg, recent TB treatment) causing individuals to test positive on Xpert Ultra would likely also cause them to test positive on the less specific screening tests, we assumed that all individuals who would test false-positive with Xpert Ultra would also test false positive on the screening test. We relaxed this assumption of conditional independence between screening and confirmatory testing in the sensitivity analysis.

The model was parameterized with country-specific information collected from the literature or using data from the R2D2 TB Network as outlined in Table 1. We benchmarked sensitivity against that of Xpert Ultra (ie, assessed sensitivity for Xpert Ultra-positive TB), as people with Xpert Ultra-negative TB would not be detected using an algorithm with Xpert Ultra as the confirmatory test. In each country, we assumed that community-based screening would take place in communities with a TB prevalence four times the estimated national average, and that 69% of prevalent TB would test positive on Xpert Ultra [5]. We estimated each country's willingness to pay using societal opportunity-cost-based thresholds [21]. In the absence of data on the societal costs of TB screening strategies, costs were estimated in 2023 United States Dollars (USD) from the healthcare system perspective. When applicable, costs were inflated to 2023 USD using each country's World Bank gross domestic product (GDP) deflator [22]. A full list of parameters is given in Table 1.

**Table 1.** List of Model Parameters by Country

Parameter	Value (Range)					Source(s)
	India	Philippines	South Africa	Uganda	Vietnam	
TB prevalence (based on Xpert-Ultra positive TB) per 100 000 screening participants	872 (800, 944)^a^	3199 (2,804, 3591)^a^	2352 (1,874, 2832)^a^	1107 (806, 1405)^a^	889 (718, 1101)^a^	[23–27]
Sensitivity of Xpert Ultra	100% (100%, 100%)^b^					[28]
Specificity of Xpert Ultra	98.8% (97.2%, 99.5%)					[5]
Sensitivity of Hypothetical Screening test	95.0% (93.4%, 96.0%)^b,c^					[13]
Specificity of Hypothetical Screening test	70.0% (63.6%, 74.5%)^b,c^					[13]
Sensitivity of CRP	64.6% (49.5%, 77.8%)^b^					STOMP-TB^d^
Specificity of CRP	84.2% (77.4%, 89.6%)^b^					STOMP-TB^d^
Proportion of screen positive people unable to sufficiently produce sputum	12.8% (0%, 26.3%)					[17]
Pre-treatment loss to follow-up	13.0% (0%, 22.1%)	9.3% (0%, 18.7%)	9.5% (0%, 20.0%)	9.5% (0%, 19.6%)	9.3% (0%, 18.7%)	[14–16, 18–20]
Cost of Hypothetical Screening test cartridge	$2.00 (fixed)					[13]
Cost of Hypothetical Screening test, total	$10.92 (8.69, 13.15)^e^	$16.96 (13.22, 20.70)^e,f^	$26.87 (24.58, 29.15)^e^	$15.49 (12.49, 21.52)^e^	$16.96 (13.22, 20.70)^e^	[8–11]
Cost of CRP test assay	$3.50 (fixed)					Boditech^g^
Cost of CRP test, total	$4.50 (4.00, 5.00)					Assumption
Cost of Xpert Ultra cartridge	$9.98 (fixed)					[29]
Cost of Xpert Ultra, total	$18.90 (16.67, 21.13)	$24.94 (21.20, 28.68)^f^	$34.85 (32.56, 37.13)	$23.47 (20.47, 29.50)	$24.94 (21.10, 28.68)	[8–11]
Cost of treatment for TB, per person	$327 (245, 409)	$392 (294, 490)	$999 (749, 1249)	$418 (314, 523)	$441 (331, 552)	[30]
WTP threshold (USD)	$560 (146, 974)	$1061 (324, 1798)	$3725 (1,486, 5963)	$192 (14, 371)	$712 (182, 1242)	[21]
DALYs averted per case detected	1.92 (1.44, 2.40)^h^					[31, 32]

Abbreviations: CRP, C-reactive protein; DALY, disability adjusted life year; R2D2, Rapid Research in Diagnostics Development TB Network; TB, tuberculosis; USD, United States dollars; WTP, willingness to pay.

^a^Assuming 4 times the national prevalence Xpert Ultra confirmed TB among adults (>15 years old).

^b^Sensitivity and specificity relative to Xpert Ultra positive samples.

^c^Using the minimal requirement of triage test target product profile (TPP), and assuming a range of ± 25%.

^d^Unpublished data from a community-based screening context.

^e^Assuming equivalent non-cartridge costs as Xpert Ultra.

^f^No cost values specific for the Philippines were available; therefore, costs equal to those in Vietnam were assumed.

^g^Personal communication with a Boditech representative.

^h^Assuming a range of the number of DALYs averted per case detected of ± 25%.

### Analysis

For each strategy and country, we simulated the model 10 000 times, with each iteration representing a distinct community-based cohort of 100 000 adults being screened for TB. In each simulation, all parameters were sampled using simple random sampling from the ranges provided in Table 1, assuming a beta distribution with the mode equal to the point estimate (further details in the Appendix, Supplementary Text 1). For the parameters where no range is given in Table 1, the point estimate was used in each simulation.

For each strategy/country, we estimated the total cost and the number of true positives, true negatives, false positives, and false negatives found at the end of all screening and confirmatory testing—as well as the cost per person screened, cost per true-positive treatment initiation, and cost per DALY averted. For the latter quantity, we assumed that 1.9 DALYs would be averted per (true positive) case detected through screening, based on a prior transmission model (using a conservative time horizon of 10 years) and accounting for the population health impact of post-tuberculosis sequelae (further details in the Appendix, Supplementary Text 2) [31, 32]. We assumed no incremental DALYs averted if people with TB were not detected (ie, false negatives) and no incremental DALYs accrued due to false-positive treatment, but we did incorporate corresponding false-positive treatment costs. We calculated incremental cost-effectiveness ratios (ICERs), defined as the incremental cost per incremental DALY averted, comparing each screening test strategy to both no case-finding and Xpert Ultra for all. We performed one-way sensitivity analyses as well as scenario analyses varying parameters and ranges as described in the Appendix (Supplementary Text 3 and SupplementaryTable 1).

For each outcome, we use the median of values observed across simulated cohorts as the point estimate. All outcomes are presented with 95% uncertainty ranges (UR), based on the 2.5th and 97.5th percentiles of values observed across the simulated cohorts. All analyses were done using R version 4.0.2. Ethical approval was not sought for this study as there was no human subject participation.

## RESULTS

Across all countries, testing the total population of 100 000 people with Xpert Ultra (universal Xpert) and assuming no losses in the care cascade was projected to avert a mean 3200 TB-related DALYs (95% UR: 2600 to 3900), at a cost of $4.0 million ($3.5 to $4.6 million). Of these costs, 64% were for testing with Xpert Ultra and the remaining 36% for treatment of people with a positive Xpert Ultra result.

Compared to universal Xpert Ultra testing, screening with point-of-care CRP was estimated to reduce costs by 54% (total cost: $1.8 million [$1.5 to $2.3 million]) but avert 44% fewer TB-related DALYs (TB-related DALYs averted: 1800 [1400 to 2300]). By contrast, using a Hypothetical Screening test (GeneXpert system-based, $2 per cartridge, 95% sensitivity for Xpert Ultra-positive TB, 70% specificity; Scenario 3a) was estimated to reduce costs by a mean of only 8% (total cost: $3.7 million [$3.2 to $4.3 million]), with 17% fewer TB-related DALYs averted (TB-related DALYs averted: 2700 [2100 to 3300]). Reducing the costs of the Hypothetical Screening test to that of point-of-care CRP ($4.50/test; Scenario 3b) was estimated to reduce costs by a mean of 41% (total cost: $2.4 million [$2.0 to $2.9]) compared to Xpert Ultra for all. Increasing the specificity of the Hypothetical Screening test to 95% (in addition to reducing its costs to those of CRP; Scenario 3c) was projected to reduce costs by a mean of 54% (total cost: $1.8 million [1.5 to 2.3]) compared to Xpert Ultra for all (Tables 2 and 3).

**Table 2.** Effectiveness of Community-based Screening for Tuberculosis Using Low-Complexity Screening Tests per 100 000 People Screened

				Median Number of DALYs Averted Through Community-Based Screening [95% UR]	
Scenario	Country	Median Number of Xpert Tests Completed [95% UR]	Median Number of People With TB Treated [95% UR]	Absolute	Relative to Xpert for All
No active case-finding—Scenario 1	All countries	0 [0, 0]	0 [0, 0]	0 [0, 0]	–
CRP—Scenario 2	India	14 000 [11000, 18 000]	430 [350, 520]	930 [720, 1200]	56%
	Philippines	15 000 [12000, 19 000]	1600 [1300, 2000]	3400 [2600, 4300]	56%
	South Africa	15 000 [11000, 19 000]	1200 [930, 1500]	2500 [1900, 3300]	56%
	Uganda	14 000 [11000, 18 000]	560 [430, 720]	1200 [860, 1600]	56%
	Vietnam	14 000 [11000, 18 000]	450 [360, 570]	950 [710, 1300]	56%
	**Mean**	**15 000 [11000, 19 000]**	**850 [670, 1100]**	**1800 [1400, 2300]**	**56%**
Hypothetical Screening test—Scenario 3a	India	27 000 [23000, 31 000]	630 [550, 720]	1400 [1100, 1700]	83%
	Philippines	28 000 [24000, 33 000]	2400 [2100, 2700]	5100 [4100, 6100]	83%
	South Africa	28 000 [24000, 32 000]	1800 [1500, 2100]	3700 [2900, 4700]	83%
	Uganda	27 000 [23000, 31 000]	820 [660, 1000]	1700 [1300, 2200]	83%
	Vietnam	27 000 [23000, 31 000]	670 [560, 800]	1400 [1100, 1800]	83%
	**Mean**	**27 000 [23000, 32 000]**	**1300 [1100, 1500]**	**2700 [2100, 3300]**	**83%**
Hypothetical Screening test—Scenario 3b	India	27 000 [23000, 31 000]	630 [550, 720]	1400 [1100, 1700]	83%
	Philippines	28 000 [24000, 32 000]	2400 [2100, 2800]	5100 [4100, 6200]	82%
	South Africa	28 000 [24000, 32 000]	1800 [1500, 2100]	3700 [2900, 4700]	83%
	Uganda	27 000 [23000, 31 000]	830 [660, 1000]	1800 [1300, 2200]	83%
	Vietnam	27 000 [23000, 31 000]	670 [560, 800]	1400 [1100, 1800]	83%
	**Mean**	**27 000 [23000, 32 000]**	**1300 [1100, 1500]**	**2700 [2100, 3300]**	**83%**
Hypothetical Screening test—Scenario 3c	India	5100 [4300, 6100]	630 [550, 720]	1400 [1100, 1700]	83%
	Philippines	6900 [6000, 8000]	2400 [2100, 2800]	5100 [4100, 6100]	83%
	South Africa	6200 [5300, 7300]	1700 [1500, 2100]	3700 [2900, 4600]	82%
	Uganda	5300 [4400, 6300]	820 [660, 1000]	1700 [1300, 2200]	83%
	Vietnam	5100 [4300, 6100]	670 [560, 790]	1400 [1100, 1800]	83%
	**Mean**	**5700 [4800, 6800]**	**1300 [1100, 1500]**	**2700 [2100, 3300]**	**83%**
Xpert for all—Scenario 4	India	100 000	760 [700, 840]	1700 [1400, 2000]	–
	Philippines	100 000	2900 [2600, 3200]	6100 [5100, 7200]	–
	South Africa	100 000	2100 [1800, 2400]	4500 [3600, 5500]	–
	Uganda	100 000	1000 [820, 1200]	2100 [1600, 2600]	–
	Vietnam	100 000	810 [700, 940]	1700 [1400, 2100]	–
	**Mean**	**100 000**	**1500 [1300, 1700]**	**3200 [2600, 3900]**	–

Figures in bold represent the mean value across all five countries analysed.

Abbreviations: CRP, C-reactive protein; DALY, disability adjusted life year; TB, tuberculosis; UR, uncertainty range.

**Table 3.** Total and Component Costs of Community-based Screening for Tuberculosis Using Low-Complexity Screening Tests per 100 000 People Screened

				Cost per Strategy Component [95% UR]					
		Total Costs per 100 000 People Screened [95% UR]		Screening		Confirmatory Testing		Treatment	
Scenario	Country	Absolute (Million USD, 2023)	Relative to Xpert for All	Absolute (Million USD, 2023)	Percent of Total Costs	Absolute (Million USD, 2023)	Percent of Total Costs	Absolute (Million USD, 2023)	Percent of Total Costs
No active case-finding—Scenario 1	All countries	0 [0, 0]	–	0 [0, 0]	–	0 [0, 0]	–	0 [0, 0]	–
CRP—Scenario 2	India	1.2 [1.0, 1.5]	47%	0.5 [0.4, 0.5]	38%	0.3 [0.2, 0.3]	23%	0.5 [0.3, 0.7]	39%
	Philippines	1.9 [1.5, 2.3]	46%	0.4 [0.4, 0.5]	24%	0.4 [0.3, 0.5]	20%	1.0 [0.7, 1.4]	55%
	South Africa	3.2 [2.5, 4.1]	47%	0.4 [0.4, 0.5]	14%	0.5 [0.4, 0.7]	16%	2.2 [1.5, 3.1]	69%
	Uganda	1.4 [1.2, 1.8]	44%	0.5 [0.4, 0.5]	31%	0.3 [0.3, 0.4]	23%	0.7 [0.4, 1.0]	45%
	Vietnam	1.5 [1.2, 1.8]	43%	0.4 [0.4, 0.5]	31%	0.4 [0.3, 0.5]	24%	0.6 [0.4, 1.0]	45%
	**Mean**	**1.8 [1.5, 2.3]**	**46%**	**0.4 [0.4, 0.5]**	**25%**	**0.4 [0.3, 0.5]**	**20%**	**1.0 [0.7, 1.5]**	**55%**
Hypothetical Screening test—Scenario 3a	India	2.1 [1.8, 2.5]	85%	1.1 [0.9, 1.2]	51%	0.5 [0.4, 0.6]	24%	0.5 [0.3, 0.8]	25%
	Philippines	3.7 [3.2, 4.3]	91%	1.7 [1.5, 1.9]	45%	0.7 [0.6, 0.8]	19%	1.3 [1.0, 1.7]	36%
	South Africa	6.4 [5.6, 7.5]	95%	2.7 [2.5, 2.8]	42%	1.0 [0.8, 1.1]	15%	2.8 [2.0, 3.8]	43%
	Uganda	3.0 [2.6, 3.5]	91%	1.6 [1.4, 1.8]	52%	0.6 [0.5, 0.8]	21%	0.8 [0.5, 1.1]	26%
	Vietnam	3.1 [2.7, 3.6]	92%	1.7 [1.5, 1.9]	54%	0.7 [0.6, 0.8]	21%	0.7 [0.5, 1.1]	24%
	**Mean**	**3.7 [3.2, 4.3]**	**92%**	**1.7 [1.6, 2.0]**	**48%**	**0.7 [0.6, 0.8]**	**19%**	**1.2 [0.9, 1.7]**	**33%**
Hypothetical Screening test—Scenario 3b	India	1.5 [1.3, 1.8]	59%	0.4 [0.4, 0.5]	30%	0.5 [0.4, 0.6]	34%	0.5 [0.3, 0.8]	35%
	Philippines	2.5 [2.1, 2.9]	61%	0.5 [0.4, 0.5]	18%	0.7 [0.6, 0.8]	28%	1.3 [1.0, 1.7]	54%
	South Africa	4.2 [3.4, 5.2]	62%	0.4 [0.4, 0.5]	11%	1.0 [0.8, 1.1]	23%	2.8 [2.0, 3.7]	66%
	Uganda	1.9 [1.6, 2.3]	57%	0.5 [0.4, 0.5]	24%	0.6 [0.5, 0.8]	34%	0.8 [0.5, 1.1]	41%
	Vietnam	1.9 [1.6, 2.3]	55%	0.4 [0.4, 0.5]	24%	0.7 [0.6, 0.8]	36%	0.7 [0.5, 1.1]	40%
	**Mean**	**2.4 [2.0, 2.9]**	**59%**	**0.4 [0.4, 0.5]**	**19%**	**0.7 [0.6, 0.8]**	**29%**	**1.2 [0.9, 1.7]**	**52%**
Hypothetical Screening test—Scenario 3c	India	1.1 [0.9, 1.3]	43%	0.4 [0.4, 0.5]	42%	0.1 [0.1, 0.1]	9%	0.5 [0.3, 0.8]	49%
	Philippines	2.0 [1.6, 2.4]	48%	0.5 [0.4, 0.5]	23%	0.2 [0.1, 0.2]	9%	1.3 [1.0, 1.8]	68%
	South Africa	3.4 [2.7, 4.4]	51%	0.4 [0.4, 0.5]	13%	0.2 [0.2, 0.3]	6%	2.7 [2.0, 3.8]	80%
	Uganda	1.3 [1.1, 1.7]	41%	0.5 [0.4, 0.5]	33%	0.1 [0.1, 0.2]	9%	0.8 [0.5, 1.1]	57%
	Vietnam	1.3 [1.1, 1.7]	39%	0.5 [0.4, 0.5]	34%	0.1 [0.1, 0.2]	10%	0.7 [0.5, 1.1]	56%
	**Mean**	**1.8 [1.5, 2.3]**	**46%**	**0.4 [0.4, 0.5]**	**25%**	**0.1 [0.1, 0.2]**	**8%**	**1.2 [0.9, 1.7]**	**67%**
Xpert for all –Scenario 4	India	2.5 [2.2, 2.8]	–	–	0%	1.9 [1.7, 2.0]	75%	0.6 [0.4, 0.9]	25%
	Philippines	4.1 [3.6, 4.6]	–	–	0%	2.5 [2.3, 2.7]	61%	1.6 [1.2, 2.0]	39%
	South Africa	6.8 [5.9, 7.9]	–	–	0%	3.5 [3.3, 3.6]	52%	3.3 [2.5, 4.4]	48%
	Uganda	3.3 [2.9, 3.7]	–	–	0%	2.4 [2.2, 2.6]	72%	0.9 [0.6, 1.3]	28%
	Vietnam	3.4 [3.0, 3.8]	−	−	0%	2.5 [2.3, 2.7]	74%	0.9 [0.6, 1.3]	26%
	**Mean**	**4.0 [3.5, 4.6]**	**−**	**−**	**0%**	**2.5 [2.4, 2.8]**	**64%**	**1.5 [1.1, 2.0]**	**36%**

Figures in bold represent the mean value across all five countries analysed.

Abbreviations: CRP, C-reactive protein; UR, uncertainty range; USD, United States dollars.

The costs of screening, confirmatory testing and treatment relative to the total costs of the active case-finding intervention were similar across countries (Appendix, Supplementary Table 2). However, for the baseline hypothetical screening test (Scenario 3a) and universal Xpert Ultra (Scenario 4), testing was the largest cost component, whereas treatment represented the majority of total costs for the CRP strategy (Scenario 2) and the improved hypothetical screening tests (Scenarios 3b-c) (Table 3).

### Incremental Cost-effectiveness

**Figure 1.**
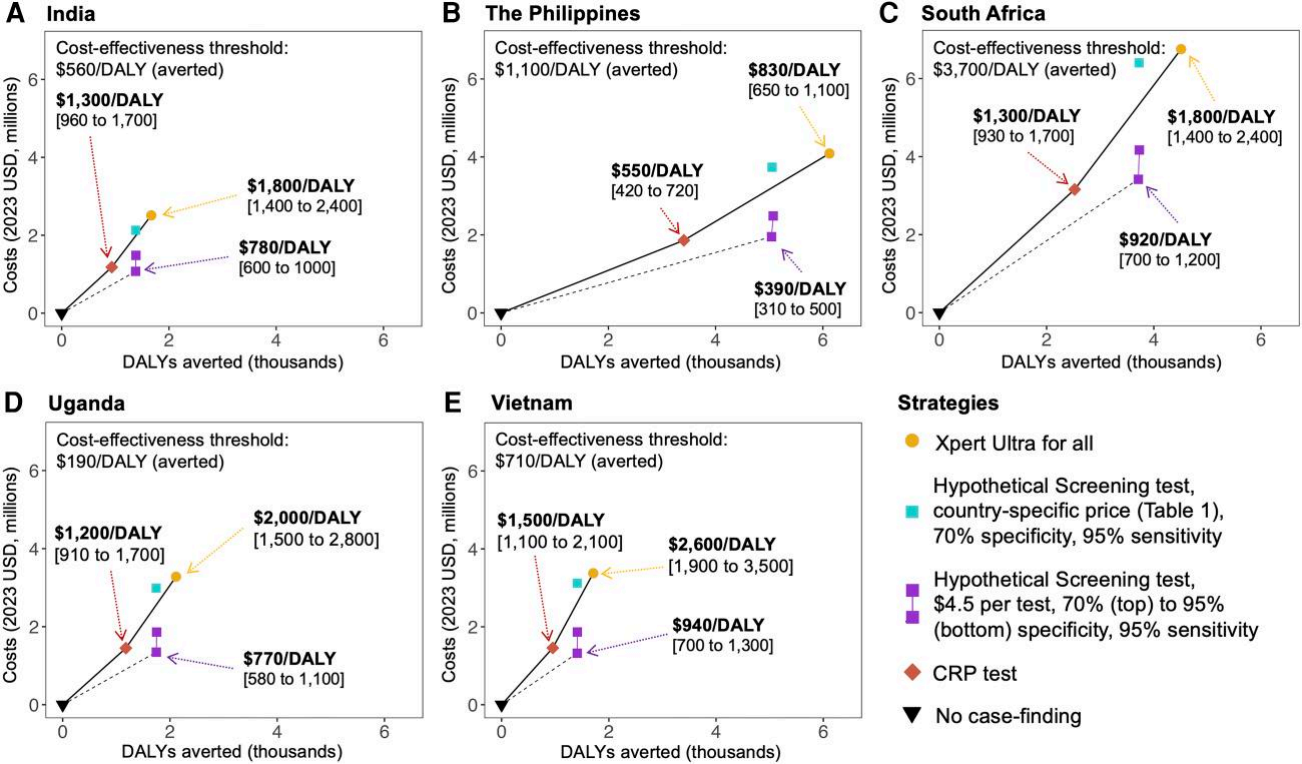
Efficiency frontiers for different community-based screening approaches for tuberculosis. Panels A to E show total costs in 2023 (USD, y-axis, in millions) of each screening strategy plotted against the number of DALYs averted (x-axis, in thousands), per 100 000 people screened. The black triangle corresponds to case-finding Scenario 1 (no case-finding, with further details in the methods), the red square to Scenario 2 (CRP testing), and the yellow circle to Scenario 4 (Xpert for all). The different scenarios (3a–c) are denoted by the blue and purple squares: the single blue square (top) corresponds to Scenario 3a, the upper of the 2 purple squares to Scenario 3b, and the lower purple square to Scenario 3c. The slope of the solid line represents the ICER of scenarios 1, 2, and 3a; that is, the cost per DALY averted upon shifting from 1 strategy to the next most effective strategy along the efficiency frontier. Strategies not on the frontier are less cost-effective than combinations of strategies that appear on the frontier, and only the ICERs between those strategies appearing on the frontier are shown. For example, in India (panel A), the CRP (Scenario 2, yellow), the Hypothetical Screening test (Scenario 3a, blue), and the Xpert for all (Scenario 1, yellow) strategy are on the efficiency frontier, and respective ICERs are noted next to each strategy. In the other countries, the baseline Hypothetical Screening test is always less cost-effective than Xpert for all, and only the ICER comparing Xpert for all to CRP is shown. The ICER resulting from an improved Hypothetical Screening test (Scenario 3c, lower purple square) is shown by the dotted black line. Supplementary Figure 5 presents an alternative analysis for South Africa, reflecting alternative, lower cost estimates in that setting. Abbreviations: CRP, C-reactive protein; DALY, disability adjusted life year; ICER, incremental cost-effectiveness ratio, USD, United States dollars.

Compared to no case-finding, Xpert Ultra for all was estimated to cost between $670/DALY averted (95% UR: $550 to $830) (The Philippines) to $2000/DALY averted ($1600 to $2500) (Vietnam). Screening with the Hypothetical Screening test (Scenario 3a) was associated with marginally higher costs per DALY averted: $740/DALY averted ($600 to $930) (The Philippines) to $2200/DALY averted ($1700 to $2900) (Vietnam). Screening with point-of-care CRP involved modestly lower costs per DALY averted ($550/DALY averted [$420 to $720] in The Philippines to $1500/DALY averted [$1100 to $2100] in Vietnam). If a screening test could be developed with 95% sensitivity and specificity for Xpert-positive TB and a fully loaded cost of $4.50 per test (Scenario 3c), estimated costs per DALY averted were lower, from $390/DALY averted ($310 to $500) in The Philippines to $940/DALY averted ($700 to $1300) in Vietnam (Figure 1). Further details are given in the Appendix (Supplementary Table 3).

**Figure 2.**
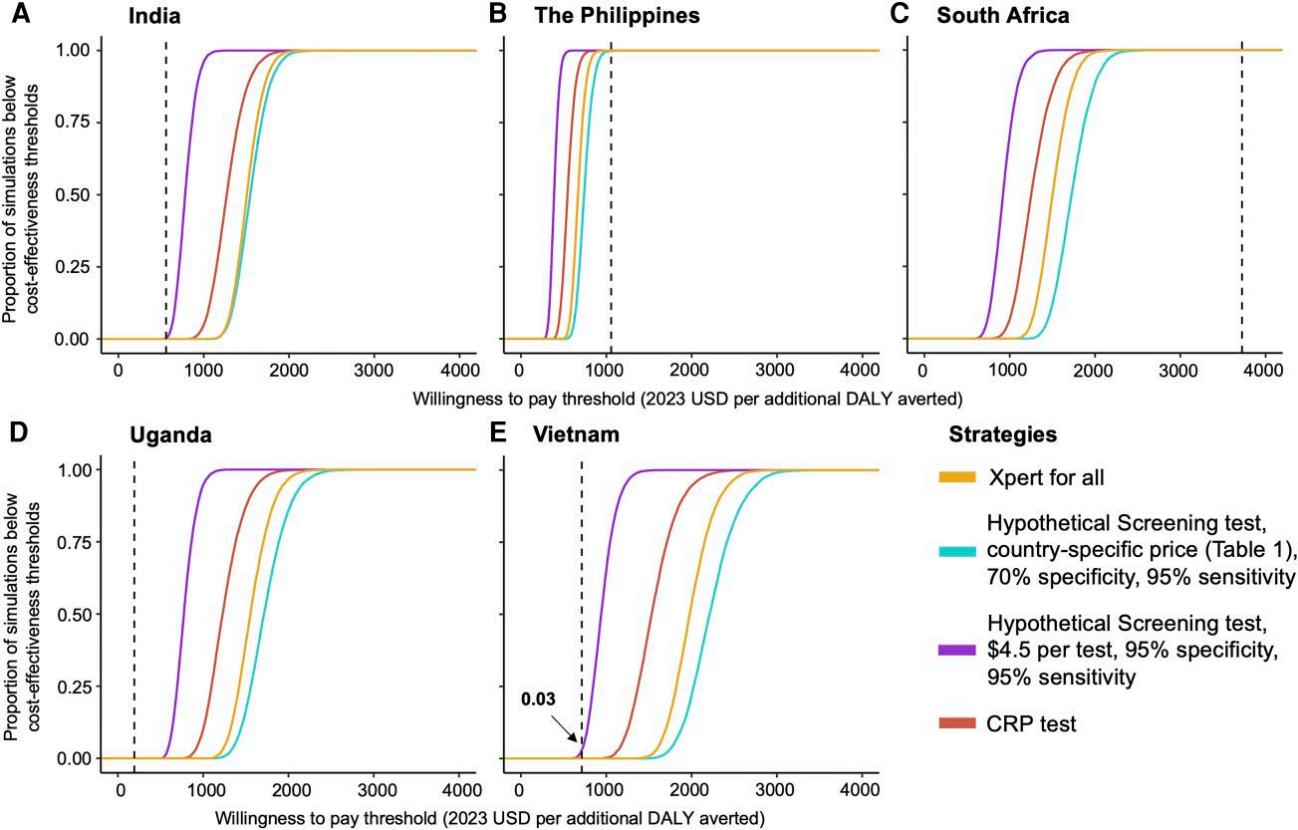
Cost-effectiveness acceptability curves for community-based tuberculosis screening strategies in three countries. The y-axis of each graph shows the percentage of simulations falling below the willingness-to-pay threshold presented on the x-axis, for case-finding Scenario 2 (CRP test, red line), Scenario 3a (Hypothetical Screening test with country-specific costs, 95% sensitivity compared to Xpert Ultra positive TB, and 70% specificity; blue line), Scenario 3c (improved Hypothetical Screening test costing $4.5 per test, 95% sensitivity compared to Xpert Ultra positive TB, and 95% specificity; purple line) and Scenario 4 (Xpert for all; yellow line). As shown in Table 1, the prevalence of TB in India, Vietnam, and Uganda is lower than in South Africa or The Philippines. Corresponding country-specific willingness-to-pay thresholds [21] are represented as a vertical dotted red line in each graph. These thresholds correspond with economic development and are thus highest in South Africa, intermediate in The Philippines, and lowest in India, Uganda, and Vietnam. Labels denote the percentage of simulations falling below this willingness-to-pay threshold. Abbreviations: CRP, C-reactive protein; DALY, disability adjusted life year; TB, tuberculosis; USD, United States dollars.

In South Africa and The Philippines (the 2 countries with the highest TB prevalence and willingness-to-pay thresholds), community-based case-finding was broadly cost-effective, with 100% of simulations estimating that any of the case-finding strategies would be below the country-specific willingness-to-pay threshold. On the contrary, in India, Vietnam, and Uganda, community-based case-finding was unlikely to be cost-effective in communities with only four-fold higher TB prevalence than national estimates (Figure 2). Estimates of the price points at which screening assays could reach cost-effectiveness thresholds can be found in the Appendix (Supplementary Table 4).

**Figure 3.**
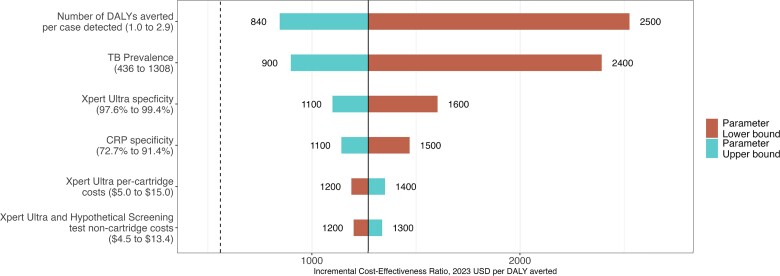
One-way sensitivity analysis on the incremental cost-effectiveness of CRP testing versus no case-finding for tuberculosis in India. Shown is the ICER (x-axis) of screening with CRP test for TB in India, relative to no case-finding, under one-way variation of key model parameters (range given below each parameter, baseline values are presented in Table 1). Parameters for which variation did not change ICER estimates by more than ±10% are not shown. The numbers to the left and right of each bar show the high and low values of the ICER (rounded to 2 significant digits) when varying the respective parameter (blue bars = upper bound of the parameter's range, red bars = lower bound of the parameter's range [there is no lower bound for loss to follow-up, as the loss to follow-up was already at 0% in the baseline model]). The black vertical line specifies the ICER ($1300 per DALY averted in Figure 1) when all parameters are held at their baseline values. The dashed vertical line represents India's willingness-to-pay ($560 per DALY averted). Abbreviations: CRP, C-reactive protein; DALY, disability adjusted life year; ICER, incremental cost-effectiveness ratio; TB, tuberculosis.

In 1-way sensitivity analysis (that does not incorporate the relative uncertainty in each parameter value), cost-effectiveness of screening varied most with TB prevalence and the number of DALYs averted per TB case detected (Figure 3). Further sensitivity and scenario analyses are provided in the Appendix (Supplementary Text 4, Supplementary Tables 5–7, and Supplementary Figures 1–5).

## DISCUSSION

This model-based analysis of the effectiveness and cost-effectiveness of community-based TB screening strategies found that low-complexity screening tools can achieve meaningful improvements in TB diagnosis but only if they are highly sensitive, highly specific (approximately 70%–95%), and available at low cost (approaching $4.5/test all-inclusive). These results can help frame future development and implementation of TB screening tests for community-based active case finding in high-burden settings.

Our results illustrate some of the challenges in developing screening tests for purposes of reducing the cost of systematic TB screening, even in high prevalence settings (4 times the national prevalence of representative high-burden countries). Compared to universal Xpert Ultra testing, using a high-sensitivity test with modest specificity (70%) that requires infrastructure such as the GeneXpert platform, for example, Hypothetical Screening Test (Scenario 3a), was estimated to result in almost no cost savings. This reflects both the high infrastructure costs of such a screening test, for example, the cost of GeneXpert equipment, and the cost of confirmatory testing for 30% of the population (1—specificity of 70%). By contrast, point-of-care CRP—with low per-test cost ($4.50 fully loaded) and somewhat higher specificity—could cut the costs of case-finding in half. However, this lower overall cost would come at the expense of a 44% reduction in the effectiveness of the case-finding, arising from the low sensitivity of CRP in the community-based testing context. Still, if a highly sensitive and specific screening test with low costs could be developed (eg, combining the advantages of the CRP and Hypothetical Screening test, Scenario 3c), screening tests could meaningfully reduce the costs per DALY averted of community-based case-finding.

Importantly, this analysis shows that the cost-effectiveness of TB case-finding depends not only on the accuracy and cost of any screening test used but also on TB prevalence (in the screened population), outcomes in people with TB who are missed by the health system, and willingness to pay. A substantial portion of the costs of case-finding—even with universal Xpert Ultra—reflect the costs of treating the people with Xpert-positive TB whom it identifies. Thus, in settings where willingness-to-pay is similar to the cost-effectiveness of TB treatment alone (ie, with no screening costs), even a perfect screening test will not be sufficient to achieve cost-effectiveness. Consequently, our analysis found case-finding to be cost-effective compared to no case-finding only in South Africa and The Philippines, representing these countries’ comparably high income and TB prevalence. Also, 1-way sensitivity analyses suggested that the strongest drivers of cost-effectiveness were TB prevalence and the number of DALYs averted per case detected—further illustrating the importance of performing screening in populations with high TB prevalence and high likelihood of adverse outcomes from missed or delayed TB diagnosis (eg, due to poor access to care).

Previous analyses have estimated that use of a screening test could reduce case-finding costs by 30% to 50% without significant reductions in DALYs averted compared to Xpert for all [33, 34]—somewhat higher than our estimates based on realistic screening assays. However, these prior studies assumed screening tools to be more accurate than CRP and less expensive than the Hypothetical Screening test (using the GeneXpert platform plus costs of $2 per cartridge), suggesting that earlier estimates of the cost-effectiveness of screening tools may have been optimistic relative to low-complexity screening options that are currently available (or likely to be available in the near future).

Our model is limited in that it is a highly simplified representation of community-based case-finding. It does not consider characteristics such as human immunodeficiency virus (HIV) or symptom status, or bacillary load, which can affect the accuracy of the evaluated diagnostics and alter the individual and population health benefits resulting from early detection [32]. Including these characteristics might change the effectiveness and cost-effectiveness results found here. Furthermore, our model does not explicitly consider outcomes among individuals with prevalent TB who would otherwise present for routine care. In addition to these simplifications, assessments of cost-effectiveness were highly dependent on willingness-to-pay thresholds, which are poorly characterized (especially in lower-income settings) [35]. We based our cost-effectiveness thresholds on recent guidance [36], but we used thresholds based on a societal perspective without including costs (or savings) outside of the healthcare sector. Furthermore, our costs of Xpert Ultra and TB treatment may be overestimated in some settings (particularly South Africa) [37, 38]. More expensive screening strategies might therefore have a higher probability of cost-effectiveness assuming higher classical thresholds (eg, based on per capita GDP) or lower estimated diagnostic/treatment costs, but a lower probability of cost-effectiveness under lower revealed thresholds or with high patient (or other non-healthcare-sector) costs of TB screening and treatment [39]. Finally, we assumed that the screening tools analyzed would only be performed in settings where chest X-ray is not available. Still, chest X-ray has characteristics similar to the most optimal diagnostics analyzed here (95% sensitivity, 80%–90% specificity, costs per screen of <$2 when performing a sufficiently large number of screens). Thus, where introduction would be feasible given infrastructure and screening volumes, chest X-ray is likely to be a very useful screening tool.

In conclusion, this model-based analysis of community-based screening for TB found that low-complexity screening tests can improve the cost-effectiveness of community-based case-finding for TB but only if they are sensitive, highly specific, and inexpensive. These findings suggest that further improvement of screening tests and a more detailed understanding of their potential use cases is required before the large-scale use of screening tests can be recommended on economic grounds. Future work should explore the cascade of diagnosis and implementation of different screening modalities under real-world conditions.

## Supplementary Data

Supplementary materials are available at *Clinical Infectious Diseases* online. Consisting of data provided by the authors to benefit the reader, the posted materials are not copyedited and are the sole responsibility of the authors, so questions or comments should be addressed to the corresponding author.

## Supplementary Material

ciad501_Supplementary_Data

## Notes

***Author contributions.*** S. S., E. A. K., A. C., C. M. D., and D. W. D. conceived the study. L. E. B., R. T., and A. M. wrote the first draft and performed initial analyses. L. E. B. and D. W. D. further refined the manuscript and analysis based on additional comments. All authors provided comments on the manuscript and analyses and approved the final version for submission.

***Acknowledgments.*** The authors acknowledge support from the Else Kröner-Fresenius-Stiftung within the Heidelberg Graduate School of Global Health and from the German Academic Exchange Foundation.

***Financial support*.** This work was supported by the German Center for Infection Research (grant number 80295MD001 to L. E. B. and grant number 8029802812 to C. M. D.) and the US National Institutes of Health (NIH) (grant number U01AI152087 to S. S., A. C., and C. M. D., grant number R01HL138728 to D. W. D. and E. A. K., and grant number R01HL153611 to E. A. K.). P. N. reports the following support for this work: NIH National Institute of Allergy and Infectious Diseases, Continuation, A135696, FEND Contract (R2D2 TB Network).
