# Supplementary material for: Cost-effectiveness of Low-complexity Screening Tests in Community-based Case-finding for Tuberculosis
Source: Clin Infect Dis. 2023 Aug 25;78(1):154–63. doi: 10.1093/cid/ciad501 (PMC10810711; doi:10.1093/cid/ciad501)
Supplement: ciad501_Supplementary_Data [file ciad501_supplementary_data.docx]

APPENDIX

Table of Contents

[Text S1. Parameter sampling for Monte Carlo simulation. 2](#_Toc140212326)

[Text S2. Estimation of the number of DALYs averted per cases detected. 3](#_Toc140212327)

[Text S3. Methods for sensitivity analysis 4](#_Toc140212328)

[Text S4. Supplementary Results. 5](#_Toc140212329)

[Table S1. Parameter ranges for one-way sensitivity analysis. 7](#_Toc140212330)

[Table S2. Detailed costs of each screening strategy per country. 9](#_Toc140212331)

[Table S3. Incremental cost-effectiveness of each of the case-finding strategies. 13](#_Toc140212332)

[Table S4. Target price points for each screening strategy per country. 14](#_Toc140212333)

[Table S5. Incremental cost-effectiveness of each case-finding strategy relaxing the assumption that every person testing false positive on Xpert Ultra also tests false positive on the screening tests. 15](#_Toc140212334)

[Table S6. Incremental cost-effectiveness of screening compared to no case finding under varying non-cartridge Xpert Ultra and treatment costs. 16](#_Toc140212335)

[Table S7. Incremental cost-effectiveness of each case-finding strategy assuming all parameters to follow a uniform distribution. 18](#_Toc140212336)

[Figure S1. One-way sensitivity analysis on the incremental cost-effectiveness of CRP testing versus no screening for tuberculosis. 19](#_Toc140212337)

[Figure S2. One-way sensitivity analysis on the incremental cost-effectiveness of the Hypothetical Screening test versus no screening for tuberculosis. 21](#_Toc140212338)

[Figure S3. Efficiency frontiers for different community-based screening approaches for tuberculosis in Uganda including screening with chest X-ray and symptom screen. 23](#_Toc140212339)

[Figure S4. Scenario analysis: efficiency frontiers for CRP screening in an HIV-positive clinic-based population. 25](#_Toc140212340)

[Figure S5. Scenario analysis: efficiency frontiers for different community-based screening approaches for tuberculosis in South Africa using lowest Xpert Ultra and treatment cost estimates. 26](#_Toc140212341)

# Text S1. Parameter sampling for Monte Carlo simulation.

The ranges for sampling each parameter are listed in Table 1. We assumed each parameter to follow a beta distribution, with the mode equal to the parameter’s point estimate. Where the upper and the lower range of a parameter were not equal to 1 and 0, respectively, we first used the beta distribution to randomly sample a value (“y”) between 0 and 1. Afterwards, we calculated the corresponding value of the parameter for each simulation using the following formula:

x_sampled_ = x_min_ + (x_max_ – x_min_) * y,

with x_sampled_ representing the value of the randomly sampled parameter, x_min_ the lower range of the parameter, x_max_ the upper range of the parameter, and y the randomly sampled value from the beta distribution (between 0 and 1). We assumed alpha = 4 for all parameters (which corresponds to a 95% confidence interval covering 63% of the width of the full distribution, when the beta distribution was symmetric). As a separate sensitivity analysis, we assumed all parameters to follow a uniform distribution, which had minimal effect on our primary results (Table S7).

# Text S2. Estimation of the number of DALYs averted per cases detected.

We based the estimation of the number of DALYs averted per person with tuberculosis (TB) detected on a modeling study by Azman et al. [1]. In Figure 3 of their manuscript, Azman et al. portray the costs of a community-based case-finding strategy per DALY averted compared to the strategy’s cost per case detected for three different time horizons (two, five and ten years, discounting future health benefits with 3% per year). Considering a 10-year time horizon and a 3% yearly discount rate, across all three countries shown in the figure, one DALY averted equals approximately one case detected (i.e., [cost per DALY averted]/[cost per case detected] ~ 1).

To account for the fact that Azman et al. do not include post-TB sequelae, we also considered estimates of post-TB sequelae from Menzies et al. [2]. Menzies et al. project a total of 12.1 DALYs (95% uncertainty range [UR]: 10.0 to 14.9) suffered per person with incident TB, of which 6.3 DALYs (95% UR: 5.6 to 7.0) resulted from the actual disease, and 5.8 DALYs (95% UR: 3.8 to 8.3) from further health utility lost after successful treatment. Thus, we estimate that, for every DALY from the actual disease episode, 5.8 / 6.3 = 0.92 additional DALYs representing post-TB sequelae will be experienced.

Sources

[1] *Azman AS, Golub JE, Dowdy DW. How much is tuberculosis screening worth? Estimating the value of active case finding for tuberculosis in South Africa, China, and India. BMC Med. 2014;12:216.*

[2] *Menzies NA, Quaife M, Allwood BW, Byrne AL, Coussens AK, Harries AD, et al. Lifetime burden of disease due to incident tuberculosis: a global reappraisal including post-tuberculosis sequelae. Lancet Glob Health. 2021;9(12):e1679-e87.*

# Text S3. Methods for sensitivity analysis

Parameters were varied by +/- 50%, except for loss to follow-up (LTFU) between screening and Xpert Ultra testing and pre-treatment LTFU, which was modeled to be 0% in the respective sensitivity analysis. For parameters that represent proportions, we first converted those values to odds, which were varied by +/- 50% before conversion back to the arithmetic scale (to prevent values <0 or >1.0). By assuming similar ranges across all parameters, this approach increases transparency and reproducibility. This analysis therefore provides results akin to elasticities, but does not account for the relative level of uncertainty across parameters. To the extent that certain parameter values are more uncertain, they may have more influence over the results of this analysis. Table S1 provides the ranges for all parameters in the one-way sensitivity analysis. In addition, we evaluated the effect of relaxing the assumption that all adults testing false positive on Xpert Ultra would also test positive on any of the screening tests and benchmarking Xpert Ultra and treatment cost estimates to local studies (instead of more generalizable values used in the main analysis).

# Text S4. Supplementary Results.

Tables S2 and S3 provide details of the itemized costs of each intervention in each country and corresponding estimates of incremental cost-effectiveness, respectively. While the ability to cross cost-effectiveness thresholds was not a main outcome of this analysis, we also estimated (in Table S4) the price points at which screening with CRP and the Hypothetical Screening test would be considered cost-effective compared to no case-finding and universal Xpert Ultra, assuming country-specific cost-effectiveness thresholds [1].

Under one-way sensitivity analysis, the cost-effectiveness of screening with either CRP or the Hypothetical Screening test (versus no case-finding) was most sensitive to the number of DALYs averted per case detected and the TB prevalence in the screened population, followed by the specificity of Xpert Ultra confirmatory testing (Figure 3 in the main manuscript; Figures S1 and S2 below).

Additional scenario analyses are shown in Table S5, Figure S3, and Figure S4. Relaxing the assumption that all adults testing false positive on Xpert Ultra would also test positive on any of the screening tests (i.e., that first-pass screening would not reduce the number of false-positive tests) improved the cost-effectiveness of screening strategies (Table S5). Figure S3 presents a scenario analysis in which symptom screening and chest X-ray are also considered as potential screening strategies; these strategies appeared close to or on the efficiency frontier but did not substantially alter our primary findings regarding low-complexity assays. Figure S4 shows the costs and effect of CRP as it might be found in an HIV-positive clinic-based population, assuming CRP to be 96% sensitive for Xpert Ultra positive TB and 12% specific [2] - in contrast to the main analysis’ assumption of 65% sensitivity for Xpert Ultra positive TB and 84% specificity in a community-based screening context [3] (all other parameters were hold constant). While in the HIV-positive clinic-based setting CRP is projected to avert nearly as many DALYs as universal Xpert Ultra, due to its low specificity, it produces costs higher than universal Xpert Ultra in almost all countries.

Table S6 presents a multivariable sensitivity analysis to evaluate the degree to which incremental cost-effectiveness estimates depend on the cost of Xpert Ultra testing and TB treatment in each country. In this analysis, all variables presented in Table 1 (main manuscript text) are varied over a wider range, with the lower bound of Xpert non-cartridge costs assumed to be 20% that in the primary analysis (based on [4, 5] from South Africa) and the lower bound of treatment costs assumed to be 11% that in the primary analysis (based on [6] in South Africa). Also, we induced a correlation of 1.0 between Xpert Ultra non-cartridge costs and TB treatment costs (reflecting that biases affecting one of these parameters might reasonably affect the other as well). We then present the estimated incremental cost-effectiveness for each screening strategy according to the decile of Xpert non-cartridge costs + treatment costs, across 10,000 simulations. Variation in these estimated costs generally resulted in variation of the estimated incremental cost-effectiveness ratio by approximately a factor of two, comparing simulations in the lowest decile of costs to simulations in the highest decile; this variation was greatest in South Africa (where these costs were highest) and lowest in India. Efficiency frontiers corresponding to the lowest assumed costs in South Africa are shown in Figure S5.

Sources

[1] *Woods B, Revill P, Sculpher M, Claxton K. Country-Level Cost-Effectiveness Thresholds: Initial Estimates and the Need for Further Research. Value Health. 2016;19(8):929-35.*

[2] *Dhana A, Hamada Y, Kengne AP, Kerkhoff AD, Rangaka MX, Kredo T, et al. Tuberculosis screening among HIV-positive inpatients: a systematic review and individual participant data meta-analysis. Lancet HIV. 2022;9(4):e233-e41.*

[3] *yet unpublished data from the STOMP-TB study*

[4] *Kgowedi S, Girdwood S, Govender K, Lekodeba N, Meyer-Rath G, Miot J, et al. Cost and outcomes of drug susceptible TB treatment at primary healthcare clinics. Johannesburg; 2020.*

[5] *Cassim N, Coetzee LM, Makuraj AL, Stevens WS, Glencross DK. Establishing the cost of Xpert MTB/RIF mobile testing in high-burden peri-mining communities in South Africa. Afr J Lab Med. 2021;10(1):1229*

[6] *Meyer-Rath G, van Rensburg C, Larson B, Jamieson L, Rosen S. Revealed willingness-to-pay versus standard cost-effectiveness thresholds: Evidence from the South African HIV Investment Case. PLoS One. 2017;12(10):e0186496*

# Table S1. Parameter ranges for one-way sensitivity analysis.

|  | | Baseline | | | | | Decrease | | | | | Increase | | | | |
| --- | --- | --- | --- | --- | --- | --- | --- | --- | --- | --- | --- | --- | --- | --- | --- | --- |
|  |  | India | The Philip-pines | South Africa | Uganda | Viet-nam | India | The Philip-pines | South Africa | Uganda | Viet-nam | India | The Philip-pines | South Africa | Uganda | Viet-nam |
| 1) TB Prevalence per 100,000; increase and decrease by 50% each | Point estimate | 872 | 3199 | 2352 | 1107 | 889 | 436 | 1599 | 1176 | 553 | 445 | 1308 | 4799 | 3528 | 1661 | 1334 |
|  | Lower range | 800 | 2804 | 1874 | 806 | 718 | 400 | 1402 | 937 | 403 | 359 | 1200 | 4206 | 2811 | 1209 | 1077 |
|  | Upper range | 944 | 3591 | 2832 | 1405 | 1101 | 472 | 1795 | 1416 | 703 | 551 | 1416 | 5387 | 4248 | 2108 | 1652 |
|  |  |  |  |  |  |  |  |  |  |  |  |  |  |  |  |  |
| 2) Specificity Xpert | Point estimate | 98.8% | 98.8% | 98.8% | 98.8% | 98.8% | 97.6% | 97.6% | 97.6% | 97.6% | 97.6% | 99.4% | 99.4% | 99.4% | 99.4% | 99.4% |
|  | Lower range | 97.2% | 97.2% | 97.2% | 97.2% | 97.2% | 94.6% | 94.6% | 94.6% | 94.6% | 94.6% | 98.6% | 98.6% | 98.6% | 98.6% | 98.6% |
|  | Upper range | 99.5% | 99.5% | 99.5% | 99.5% | 99.5% | 99.0% | 99.0% | 99.0% | 99.0% | 99.0% | 99.7% | 99.7% | 99.7% | 99.7% | 99.7% |
|  |  |  |  |  |  |  |  |  |  |  |  |  |  |  |  |  |
| 3) No LTFU due to inability to sufficiently produce sputum | Point estimate | 12.8% | 12.8% | 12.8% | 12.8% | 12.8% | 0% | 0% | 0% | 0% | 0% | -- | -- | -- | -- | -- |
|  | Lower range | 0% | 0% | 0% | 0% | 0% | 0% | 0% | 0% | 0% | 0% | -- | -- | -- | -- | -- |
|  | Upper range | 26.3% | 26.3% | 26.3% | 26.3% | 26.3% | 0% | 0% | 0% | 0% | 0% | -- | -- | -- | -- | -- |
|  |  |  |  |  |  |  |  |  |  |  |  |  |  |  |  |  |
| 3) No pre-treatment LTFU | Point estimate | 13.0% | 9.3% | 9.5% | 9.5% | 9.3% | 0% | 0% | 0% | 0% | 0% | -- | -- | -- | -- | -- |
|  | Lower range | 0% | 0% | 0% | 0% | 0% | 0% | 0% | 0% | 0% | 0% | -- | -- | -- | -- | -- |
|  | Upper range | 22.1% | 18.7% | 20.0% | 19.6% | 18.7% | 0% | 0% | 0% | 0% | 0% | -- | -- | -- | -- | -- |
|  |  |  |  |  |  |  |  |  |  |  |  |  |  |  |  |  |
| 4) Cost of Xpert Ultra cartridge; increase and decrease by 50% each | Point estimate | 9.98 | 9.98 | 9.98 | 9.98 | 9.98 | 4.99 | 4.99 | 4.99 | 4.99 | 4.99 | 14.97 | 14.97 | 14.97 | 14.97 | 14.97 |
|  | Lower range | 9.98 | 9.98 | 9.98 | 9.98 | 9.98 | 4.99 | 4.99 | 4.99 | 4.99 | 4.99 | 14.97 | 14.97 | 14.97 | 14.97 | 14.97 |
|  | Upper range | 9.98 | 9.98 | 9.98 | 9.98 | 9.98 | 4.99 | 4.99 | 4.99 | 4.99 | 4.99 | 14.97 | 14.97 | 14.97 | 14.97 | 14.97 |
|  |  |  |  |  |  |  |  |  |  |  |  |  |  |  |  |  |
| 5) Xpert Ultra and Hypothetical Screening test non-cartridge costs; increase and decrease by 50% each | Point estimate | 18.90 | 24.94 | 34.85 | 23.47 | 24.94 | 14.44 | 17.46 | 22.41 | 16.72 | 17.46 | 23.36 | 32.42 | 47.29 | 30.22 | 32.42 |
|  | Lower range | 16.67 | 21.20 | 32.56 | 20.47 | 21.10 | 13.33 | 15.59 | 21.27 | 15.23 | 15.59 | 20.02 | 26.81 | 43.85 | 25.72 | 26.81 |
|  | Upper range | 21.13 | 28.68 | 37.13 | 29.50 | 28.68 | 15.56 | 19.33 | 23.56 | 19.74 | 19.33 | 26.71 | 38.03 | 50.71 | 39.26 | 38.03 |
|  |  |  |  |  |  |  |  |  |  |  |  |  |  |  |  |  |
| 6) Cases detected to DALYs averted conversion rate; increase and decrease by 50% each | Point estimate | 1.92 | 1.92 | 1.92 | 1.92 | 1.92 | 0.96 | 0.96 | 0.96 | 0.96 | 0.96 | 2.88 | 2.88 | 2.88 | 2.88 | 2.88 |
|  | Lower range | 1.44 | 1.44 | 1.44 | 1.44 | 1.44 | 0.72 | 0.72 | 0.72 | 0.72 | 0.72 | 2.16 | 2.16 | 2.16 | 2.16 | 2.16 |
|  | Upper range | 2.40 | 2.40 | 2.40 | 2.40 | 2.40 | 1.20 | 1.20 | 1.20 | 1.20 | 1.20 | 3.60 | 3.60 | 3.60 | 3.60 | 3.60 |
|  |  |  |  |  |  |  |  |  |  |  |  |  |  |  |  |  |
| 7) CRP test specificity | Point estimate | 0.84 | 0.84 | 0.84 | 0.84 | 0.84 | 0.73 | 0.73 | 0.73 | 0.73 | 0.73 | 0.91 | 0.91 | 0.91 | 0.91 | 0.91 |
|  | Lower range | 0.77 | 0.77 | 0.77 | 0.77 | 0.77 | 0.63 | 0.63 | 0.63 | 0.63 | 0.63 | 0.87 | 0.87 | 0.87 | 0.87 | 0.87 |
|  | Upper range | 0.90 | 0.90 | 0.90 | 0.90 | 0.90 | 0.81 | 0.81 | 0.81 | 0.81 | 0.81 | 0.95 | 0.95 | 0.95 | 0.95 | 0.95 |

CRP, C-reactive protein; LTFU, loss to follow up

# Table S2. Detailed costs of each screening strategy per country.

|  | | Cost **per person screened** (2023 USD) | | | | | | |
| --- | --- | --- | --- | --- | --- | --- | --- | --- |
|  |  | Screening | | Confirmatory testing | | Treatment | | Total |
|  |  | Absolute [95% UR] | Relative | Absolute [95% UR] | Relative | Absolute [95% UR] | Relative | Absolute [95% UR] |
| CRP –  Scenario 2 | India | 4 [4, 5] | 38% | 3 [2, 3] | 23% | 5 [3, 7] | 39% | 12 [10, 15] |
|  | Philippines | 4 [4, 5] | 24% | 4 [3, 5] | 20% | 10 [7, 14] | 55% | 19 [15, 23] |
|  | South Africa | 4 [4, 5] | 14% | 5 [4, 7] | 16% | 22 [15, 31] | 69% | 32 [25, 41] |
|  | Uganda | 4 [4, 5] | 31% | 3 [3, 4] | 23% | 7 [4, 10] | 45% | 14 [12, 18] |
|  | Vietnam | 4 [4, 5] | 31% | 4 [3, 5] | 24% | 6 [4, 10] | 44% | 15 [12, 18] |
|  | **Mean** | **4 [4, 5]** | **25%** | **4 [3, 5]** | **20%** | **10 [7, 15]** | **55%** | **18 [15, 23]** |
| Hypothetical Screening test –  Scenario 3a | India | 11 [10, 12] | 51% | 5 [4, 6] | 24% | 5 [3, 8] | 25% | 21 [18, 25] |
|  | Philippines | 17 [15, 19] | 45% | 7 [6, 8] | 19% | 13 [10, 17] | 36% | 37 [32, 43] |
|  | South Africa | 27 [25, 28] | 42% | 10 [8, 11] | 15% | 28 [20, 38] | 43% | 64 [56, 75] |
|  | Uganda | 16 [14, 18] | 52% | 6 [5, 8] | 21% | 8 [5, 11] | 26% | 30 [26, 35] |
|  | Vietnam | 17 [15, 19] | 54% | 7 [6, 8] | 21% | 7 [5, 11] | 24% | 31 [27, 36] |
|  | **Mean** | **17 [16, 20]** | **48%** | **7 [6, 8]** | **19%** | **12 [9, 17]** | **33%** | **37 [32, 43]** |
| Hypothetical Screening test –  Scenario 3b | India | 4 [4, 5] | 30% | 5 [4, 6] | 34% | 5 [3, 8] | 35% | 15 [13, 18] |
|  | Philippines | 4 [4, 5] | 18% | 7 [6, 8] | 28% | 13 [10, 17] | 54% | 25 [21, 29] |
|  | South Africa | 4 [4, 5] | 11% | 10 [8, 11] | 23% | 28 [20, 37] | 66% | 42 [34, 52] |
|  | Uganda | 4 [4, 5] | 24% | 6 [5, 8] | 34% | 8 [5, 11] | 41% | 19 [16, 23] |
|  | Vietnam | 4 [4, 5] | 24% | 7 [6, 8] | 36% | 7 [5, 11] | 40% | 19 [16, 23] |
|  | **Mean** | **4 [4, 5]** | **19%** | **7 [6, 8]** | **29%** | **12 [9, 17]** | **52%** | **24 [20, 29]** |
| Hypothetical Screening test –  Scenario 3c | India | 4 [4, 5] | 42% | 1 [1, 1] | 9% | 5 [3, 8] | 49% | 11 [9, 13] |
|  | Philippines | 4 [4, 5] | 23% | 2 [1, 2] | 9% | 13 [10, 18] | 68% | 20 [16, 24] |
|  | South Africa | 4 [4, 5] | 13% | 2 [2, 3] | 6% | 27 [20, 38] | 80% | 34 [27, 44] |
|  | Uganda | 4 [4, 5] | 33% | 1 [1, 2] | 9% | 8 [5, 11] | 57% | 13 [11, 17] |
|  | Vietnam | 4 [4, 5] | 34% | 1 [1, 2] | 10% | 7 [5, 11] | 56% | 13 [11, 17] |
|  | **Mean** | **4 [4, 5]** | **25%** | **1 [1, 2]** | **8%** | **12 [9, 17]** | **67%** | **18 [15, 23]** |
| Xpert  for all –  Scenario 4 | India | -- | -- | 19 [17, 20] | 75% | 6 [4, 9] | 25% | 25 [22, 28] |
|  | Philippines | -- | -- | 25 [23, 27] | 61% | 16 [12, 20] | 39% | 41 [36, 46] |
|  | South Africa | -- | -- | 35 [33, 36] | 52% | 33 [25, 44] | 48% | 68 [59, 79] |
|  | Uganda | -- | -- | 24 [22, 26] | 72% | 9 [6, 13] | 28% | 33 [29, 37] |
|  | Vietnam | -- | -- | 25 [23, 27] | 74% | 9 [6, 13] | 26% | 34 [30, 38] |
|  | **Mean** | **--** | **--** | **25 [24, 28]** | **64%** | **15 [11, 20]** | **36%** | **40 [35, 46]** |

|  | | Cost **per person with tuberculosis initiating treatment** (2023 USD) | | | | | | |
| --- | --- | --- | --- | --- | --- | --- | --- | --- |
|  |  | Screening | | Confirmatory testing | | Treatment | | Total |
|  |  | Absolute [95% UR] | Relative to  total costs | Absolute [95% UR] | Relative to  total costs | Absolute [95% UR] | Relative to  total costs | Absolute [95% UR] |
| CRP test –  Scenario 2 | India | 1100 [860, 1300] | 38% | 630 [460, 850] | 23% | 1100 [680, 1700] | 39% | 2800 [2200, 3600] |
|  | Philippines | 280 [220, 350] | 24% | 230 [170, 310] | 20% | 630 [480, 850] | 55% | 1200 [950, 1400] |
|  | South Africa | 380 [300, 490] | 14% | 440 [320, 590] | 16% | 1800 [1300, 2600] | 69% | 2700 [2100, 3500] |
|  | Uganda | 810 [620, 1100] | 31% | 610 [430, 870] | 23% | 1200 [760, 1800] | 45% | 2600 [2000, 3500] |
|  | Vietnam | 1000 [790, 1300] | 31% | 780 [550, 1100] | 24% | 1400 [910, 2300] | 44% | 3200 [2500, 4300] |
|  | **Mean** | **700 [560, 900]** | **28%** | **540 [390, 740]** | **22%** | **1200 [840, 1800]** | **50%** | **2500 [1900, 3300]** |
| Hypothetical Screening test –  Scenario 3a | India | 1700 [1400, 2100] | 51% | 800 [680, 950] | 24% | 830 [570, 1200] | 25% | 3400 [2900, 4000] |
|  | Philippines | 710 [570, 870] | 45% | 290 [240, 350] | 19% | 560 [440, 710] | 36% | 1600 [1300, 1800] |
|  | South Africa | 1500 [1300, 1900] | 42% | 550 [460, 670] | 15% | 1600 [1200, 2100] | 43% | 3700 [3100, 4400] |
|  | Uganda | 1900 [1500, 2500] | 53% | 780 [610, 1000] | 21% | 930 [640, 1400] | 26% | 3600 [2900, 4600] |
|  | Vietnam | 2500 [2000, 3200] | 54% | 1000 [810, 1200] | 21% | 1100 [750, 1700] | 24% | 4700 [3800, 5700] |
|  | **Mean** | **1700 [1400, 2100]** | **50%** | **680 [560, 840]** | **20%** | **1000 [720, 1400]** | **30%** | **3400 [2800, 4100]** |
| Hypothetical Screening test –  Scenario 3b | India | 710 [610, 830] | 30% | 800 [680, 950] | 34% | 840 [560, 1200] | 35% | 2400 [2000, 2800] |
|  | Philippines | 190 [160, 220] | 18% | 290 [240, 350] | 28% | 560 [440, 710] | 53% | 1000 [900, 1200] |
|  | South Africa | 260 [210, 310] | 11% | 550 [460, 670] | 23% | 1600 [1200, 2100] | 66% | 2400 [2000, 2900] |
|  | Uganda | 550 [440, 690] | 24% | 770 [610, 1000] | 34% | 930 [640, 1400] | 41% | 2300 [1800, 2900] |
|  | Vietnam | 670 [560, 820] | 24% | 1000 [810, 1200] | 36% | 1100 [750, 1700] | 40% | 2800 [2300, 3500] |
|  | **Mean** | **480 [400, 580]** | **22%** | **680 [560, 840]** | **32%** | **1000 [720, 1400]** | **46%** | **2200 [1800, 2700]** |
| Hypothetical Screening test –  Scenario 3c | India | 710 [610, 840] | 42% | 150 [130, 190] | 9% | 840 [560, 1200] | 49% | 1700 [1400, 2100] |
|  | Philippines | 190 [160, 220] | 23% | 72 [61, 86] | 9% | 560 [440, 710] | 68% | 820 [690, 980] |
|  | South Africa | 260 [210, 310] | 13% | 120 [110, 150] | 6% | 1600 [1200, 2100] | 80% | 2000 [1600, 2500] |
|  | Uganda | 550 [440, 690] | 33% | 150 [120, 190] | 9% | 940 [650, 1400] | 57% | 1600 [1300, 2100] |
|  | Vietnam | 680 [560, 820] | 34% | 190 [150, 240] | 10% | 1100 [750, 1700] | 56% | 2000 [1600, 2600] |
|  | **Mean** | **480 [400, 580]** | **29%** | **140 [110, 170]** | **9%** | **1000 [720, 1400]** | **62%** | **1600 [1300, 2100]** |
| Xpert  for all –  Scenario 4 | India | -- | -- | 2500 [2200, 2800] | 75% | 810 [550, 1200] | 25% | 3300 [2900, 3800] |
|  | Philippines | -- | -- | 860 [750, 990] | 61% | 550 [440, 690] | 39% | 1400 [1200, 1600] |
|  | South Africa | -- | -- | 1600 [1400, 1900] | 52% | 1500 [1200, 2000] | 48% | 3200 [2700, 3800] |
|  | Uganda | -- | -- | 2400 [1900, 3000] | 72% | 900 [630, 1300] | 27% | 3300 [2700, 4100] |
|  | Vietnam | -- | -- | 3100 [2600, 3700] | 74% | 1100 [730, 1600] | 26% | 4200 [3500, 5000] |
|  | **Mean** | **--** | **--** | **2100 [1800, 2500]** | **68%** | **980 [710, 1400]** | **32%** | **3100 [2600, 3700]** |

|  | | Cost **per DALYs averted** (2023 USD) | | | | | | |
| --- | --- | --- | --- | --- | --- | --- | --- | --- |
|  |  | Screening | | Confirmatory testing | | Treatment | | Total |
|  |  | Absolute [95% UR] | Relative to  no testing | Absolute [95% UR] | Relative to  no testing | Absolute [95% UR] | Relative to  no testing | Absolute [95% UR] |
| CRP test –  Scenario 2 | India | 480 [380, 630] | 38% | 290 [200, 400] | 23% | 490 [300, 800] | 39% | 1300 [960, 1700] |
|  | Philippines | 130 [100, 170] | 24% | 110 [80, 150] | 20% | 300 [220, 430] | 55% | 550 [420, 720] |
|  | South Africa | 180 [140, 240] | 14% | 210 [150, 290] | 16% | 860 [600, 1300] | 69% | 1300 [930, 1700] |
|  | Uganda | 380 [280, 520] | 31% | 290 [200, 420] | 23% | 560 [350, 900] | 45% | 1200 [910, 1700] |
|  | Vietnam | 470 [360, 640] | 31% | 370 [260, 530] | 24% | 680 [420, 1100] | 44% | 1500 [1100, 2100] |
|  | **Mean** | **330 [250, 440]** | **28%** | **250 [180, 360]** | **22%** | **580 [380, 900]** | **50%** | **1200 [870, 1600]** |
| Hypothetical Screening test-  Scenario 3a | India | 790 [620, 1000] | 51% | 370 [300, 460] | 24% | 380 [250, 590] | 25% | 1500 [1200, 2000] |
|  | Philippines | 340 [260, 430] | 45% | 140 [110, 180] | 19% | 260 [200, 360] | 36% | 740 [600, 930] |
|  | South Africa | 720 [570, 920] | 42% | 260 [210, 330] | 15% | 740 [540, 1000] | 43% | 1700 [1400, 2200] |
|  | Uganda | 900 [670, 1200] | 53% | 370 [280, 490] | 21% | 440 [290, 680] | 26% | 1700 [1300, 2300] |
|  | Vietnam | 1200 [910, 1600] | 54% | 470 [360, 620] | 21% | 530 [340, 820] | 24% | 2200 [1700, 2900] |
|  | **Mean** | **790 [610, 1000]** | **50%** | **320 [250, 410]** | **20%** | **470 [320, 700]** | **30%** | **1600 [1300, 2000]** |
| Hypothetical Screening test –  Scenario 3b | India | 320 [270, 410] | 30% | 370 [300, 460] | 34% | 380 [250, 580] | 36% | 1100 [860, 1400] |
|  | Philippines | 89 [72, 110] | 18% | 140 [110, 180] | 28% | 260 [200, 360] | 54% | 490 [400, 620] |
|  | South Africa | 120 [96, 160] | 11% | 260 [210, 330] | 23% | 740 [530, 1000] | 66% | 1100 [870, 1500] |
|  | Uganda | 260 [200, 340] | 24% | 370 [280, 490] | 34% | 440 [290, 680] | 41% | 1100 [820, 1400] |
|  | Vietnam | 320 [250, 410] | 24% | 470 [370, 620] | 36% | 530 [340, 820] | 40% | 1300 [1000, 1700] |
|  | **Mean** | **220 [180, 280]** | **22%** | **320 [250, 410]** | **31%** | **470 [320, 690]** | **46%** | **1000 [800, 1300]** |
| Hypothetical Screening test –  Scenario 3c | India | 330 [270, 410] | 42% | 70 [55, 89] | 9% | 380 [250, 580] | 49% | 780 [600, 1000] |
|  | Philippines | 89 [72, 110] | 23% | 34 [27, 43] | 9% | 260 [190, 360] | 68% | 390 [310, 500] |
|  | South Africa | 120 [96, 160] | 13% | 59 [47, 74] | 6% | 740 [540, 1000] | 80% | 920 [700, 1200] |
|  | Uganda | 260 [200, 340] | 33% | 72 [55, 96] | 9% | 440 [290, 670] | 57% | 770 [580, 1100] |
|  | Vietnam | 320 [250, 410] | 34% | 90 [70, 120] | 10% | 530 [340, 820] | 56% | 940 [700, 1300] |
|  | **Mean** | **220 [180, 280]** | **29%** | **65 [51, 84]** | **9%** | **470 [320, 690]** | **62%** | **760 [580, 1000]** |
| Xpert  for all –  Scenario 4 | India | -- | -- | 1100 [940, 1400] | 75% | 370 [240, 570] | 25% | 1500 [1200, 1900] |
|  | Philippines | -- | -- | 410 [330, 500] | 61% | 260 [190, 350] | 39% | 670 [550, 830] |
|  | South Africa | -- | -- | 770 [630, 970] | 51% | 730 [530, 1000] | 48% | 1500 [1200, 1900] |
|  | Uganda | -- | -- | 1100 [880, 1500] | 72% | 430 [290, 650] | 28% | 1600 [1200, 2000] |
|  | Vietnam | -- | -- | 1500 [1200, 1800] | 74% | 510 [330, 790] | 26% | 2000 [1600, 2500] |
|  | **Mean** | **--** | **--** | **980 [790, 1200]** | **68%** | **460 [320, 670]** | **32%** | **1400 [1200, 1800]** |

# Table S3. Incremental cost-effectiveness of each of the case-finding strategies.

| Scenario | Country | Willingness to pay (2023 USD per DALY averted) | Incremental cost-effectiveness ratio (2023 USD per DALY averted) | | |
| --- | --- | --- | --- | --- | --- |
|  |  |  | Vs. Xpert Ultra for all [2.5th; 97.5] | Vs. No case-finding [2.5th; 97.5] | |
|  |  |  |  | Absolute | Relative to Xpert Ultra for all |
| CRP –  Scenario 2 | India | 560 | 1800 [1400, 2400] | 1300 [960, 1700] | 84% |
|  | Philippines | 1061 | 830 [650, 1100] | 550 [420, 720] | 82% |
|  | South Africa | 3725 | 1800 [1400, 2400] | 1300 [930, 1700] | 83% |
|  | Uganda | 192 | 2000 [1500, 2800] | 1200 [910, 1700] | 79% |
|  | Vietnam | 712 | 2600 [1900, 3500] | 1500 [1100, 2100] | 77% |
|  | **Mean** | **1250** | **1800 [1400, 2400]** | **1200 [870, 1600]** | **81%** |
| Hypothetical Screening test –  Scenario 3a | India | 560 | 1300 [970, 2000] | 1500 [1200, 2000] | 103% |
|  | Philippines | 1061 | 330 [180, 470] | 740 [600, 930] | 111% |
|  | South Africa | 3725 | 440 [-87, 760] | 1700 [1400, 2200] | 115% |
|  | Uganda | 192 | 800 [430, 1200] | 1700 [1300, 2300] | 110% |
|  | Vietnam | 712 | 870 [370, 1400] | 2200 [1700, 2900] | 112% |
|  | **Mean** | **1250** | **760 [370, 1200]** | **1600 [1300, 2000]** | **110%** |
| Hypothetical Screening test –  Scenario 3b | India | 560 | 3600 [2500, 6200] | 1100 [860, 1400] | 72% |
|  | Philippines | 1061 | 1500 [1100, 2500] | 490 [400, 620] | 74% |
|  | South Africa | 3725 | 3400 [2400, 5500] | 1100 [870, 1500] | 75% |
|  | Uganda | 192 | 3900 [2600, 7000] | 1100 [820, 1400] | 69% |
|  | Vietnam | 712 | 5100 [3400, 9100] | 1300 [1000, 1700] | 67% |
|  | **Mean** | **1250** | **3500 [2400, 6100]** | **1000 [800, 1300]** | **71%** |
| Hypothetical Screening test –  Scenario 3c | India | 560 | 5000 [3300, 9200] | 780 [600, 1000] | 52% |
|  | Philippines | 1061 | 2000 [1400, 3600] | 390 [310, 500] | 58% |
|  | South Africa | 3725 | 4300 [3000, 7600] | 920 [700, 1200] | 61% |
|  | Uganda | 192 | 5300 [3400, 9800] | 770 [580, 1100] | 50% |
|  | Vietnam | 712 | 7000 [4600, 13000] | 940 [700, 1300] | 48% |
|  | **Mean** | **1250** | **4700 [3100, 8600]** | **760 [580, 1000]** | **53%** |
| Xpert Ultra  for all –  Scenario 4 | India | 560 | -- | 1500 [1200, 1900] | -- |
|  | Philippines | 1061 | -- | 670 [550, 830] | -- |
|  | South Africa | 3725 | -- | 1500 [1200, 1900] | -- |
|  | Uganda | 192 | -- | 1600 [1200, 2000] | -- |
|  | Vietnam | 712 | -- | 2000 [1600, 2500] | -- |
|  | **Mean** | **1250** | **--** | **1400 [1200, 1800]** | **--** |

N/A, not applicable (the evaluated screening strategy is dominated by the strategy compared).

# Table S4. Target price points for each screening strategy per country.

| Scenario | Country | Price point (2023 USD) to be cost-effective compared to the CRP strategy [95% UR] | | Price point (2023 USD) to be cost-effective compared to the Xpert for all strategy  [95% UR] | | Price point (2023 USD) to be cost-effective compared to no case-finding [95% UR] | |
| --- | --- | --- | --- | --- | --- | --- | --- |
|  |  | per total test | per cartridge | per total test | per cartridge | per total test | per cartridge |
| CRP test –  Scenario 2 | India | -- | -- | 14 [9, 18] | 13 [8, 17] | N/A | N/A |
|  | Philippines | -- | -- | N/A | N/A | 20 [-5, 57] | 19 [-6, 56] |
|  | South Africa | -- | -- | N/A | N/A | 59 [4, 150] | 58 [3, 150] |
|  | Uganda | -- | -- | 22 [17, 27] | 21 [16, 26] | N/A | N/A |
|  | Vietnam | -- | -- | 18 [11, 24] | 17 [10, 23] | N/A | N/A |
|  | **Mean** | **--** | **--** | **18 [12, 23]** | **17 [11, 22]** | **39 [0, 100]** | **38 [-1, 100]** |
| Hypothetical Screening test –  Scenario 3a | India | 4 [1, 7] | N/A | 13 [10, 16] | 4 [2, 7] | N/A | N/A |
|  | Philippines | 14 [3, 36] | N/A | 10 [-6, 18] | N/A | 30 [-5, 80] | 16 [-20, 65] |
|  | South Africa | 35 [10, 92] | 11 [-15, 67] | 4 [-38, 20] | N/A | 94 [13, 220] | 69 [-12, 190] |
|  | Uganda | 1 [-1, 4] | N/A | 19 [15, 24] | 4 [1, 7] | N/A | N/A |
|  | Vietnam | 4 [0, 8] | N/A | 17 [13, 22] | 2 [-1, 6] | N/A | N/A |
|  | **Mean** | **12 [3, 29]** | **11 [-15, 66]** | **13 [-1, 20]** | **3 [1, 6]** | **62 [4, 150]** | **42 [-16, 130]** |
| Xpert  for all –  Scenario 4 | India | -- | -- | -- | -- | 2 [-7, 12] | N/A |
|  | Philippines | -- | -- | -- | -- | 46 [3, 100] | 31 [-12, 90] |
|  | South Africa | -- | -- | -- | -- | 130 [29, 270] | 100 [4, 250] |
|  | Uganda | -- | -- | -- | -- | N/A | N/A |
|  | Vietnam | -- | -- | -- | -- | 2 [-10, 16] | N/A |
|  | **Mean** | **--** | **--** | **--** | **--** | **44 [4, 100]** | **67 [-4, 170]** |

N/A, not applicable (the evaluated screening strategy would not be cost-effective at country-specific willingness-to-pay thresholds in relation to its comparator even at per cartridge and / or per total test costs of $0)

# Table S5. Incremental cost-effectiveness of each case-finding strategy relaxing the assumption that every person testing false positive on Xpert Ultra also tests false positive on the screening tests.

| Scenario | Country | Willingness to pay (2023 USD per DALY averted) | Incremental cost-effectiveness ratio (2023 USD per DALY averted) | |
| --- | --- | --- | --- | --- |
|  |  |  | Vs. Xpert Ultra for all [2.5th; 97.5] | Vs. No case-finding [2.5th; 97.5] |
| CRP –  Scenario 2 | India | 560 | 2200 [1600, 3100] | 980 [770, 1300] |
|  | Philippines | 1061 | 950 [730, 1300] | 450 [360, 560] |
|  | South Africa | 3725 | 2300 [1700, 3100] | 920 [730, 1200] |
|  | Uganda | 192 | 2400 [1700, 3400] | 930 [720, 1200] |
|  | Vietnam | 712 | 3100 [2200, 4300] | 1100 [870, 1500] |
|  | **Mean** | **1250** | **2200 [1600, 3000]** | **880 [690, 1100]** |
| Hypothetical Screening test –  Scenario 3a | India | 560 | 2100 [1400, 3800] | 1400 [1100, 1700] |
|  | Philippines | 1061 | 590 [370, 1000] | 680 [560, 840] |
|  | South Africa | 3725 | 1400 [710, 2600] | 1500 [1300, 1900] |
|  | Uganda | 192 | 1600 [940, 3100] | 1500 [1200, 2000] |
|  | Vietnam | 712 | 2000 [1100, 3700] | 2000 [1600, 2500] |
|  | **Mean** | **1250** | **1500 [890, 2800]** | **1400 [1100, 1800]** |
| Xpert Ultra for all –  Scenario 1 | India | 560 | -- | 1500 [1200, 1900] |
|  | Philippines | 1061 | -- | 670 [550, 820] |
|  | South Africa | 3725 | -- | 1500 [1200, 1900] |
|  | Uganda | 192 | -- | 1600 [1200, 2000] |
|  | Vietnam | 712 | -- | 2000 [1600, 2500] |
|  | **Mean** | **1250** | **--** | **1400 [1200, 1800]** |

# Table S6. Incremental cost-effectiveness of screening compared to no case finding under varying non-cartridge Xpert Ultra and treatment costs.

| **India** (TB treatment costs ranging from $36 to $618 and  costs of Xpert Ultra from $12 to $26) | | | |
| --- | --- | --- | --- |
| **Treatment and non-cartridge Xpert Ultra cost percentile** | **Cost per DALY averted: Universal Xpert** | **Cost per DALY averted: CRP testing** | **Cost per DALY averted: Hypothetical Screening test** |
| 0 to 10^th^ | 1000 [820; 1300] | 940 [730; 1200] | 940 [680; 1200] |
| 10^th^ to 20^th^ | 1200 [1000; 1500] | 1100 [820; 1400] | 1200 [950; 1400] |
| 20^th^ to 30^th^ | 1300 [1100; 1600] | 1100 [870; 1500] | 1300 [1100; 1600] |
| 30^th^ to 40^th^ | 1400 [1200; 1700] | 1200 [920; 1600] | 1400 [1200; 1800] |
| 40^th^ to 50^th^ | 1500 [1200; 1800] | 1300 [970; 1700] | 1500 [1300; 1900] |
| 50^th^ to 60^th^ | 1600 [1300; 1900] | 1300 [1000; 1800] | 1600 [1400; 2000] |
| 60^th^ to 70^th^ | 1700 [1400; 2000] | 1400 [1000; 1800] | 1700 [1400; 2200] |
| 70^th^ to 80^th^ | 1800 [1400; 2200] | 1400 [1100; 1900] | 1900 [1500; 2300] |
| 80^th^ to 90^th^ | 1900 [1500; 2300] | 1500 [1200; 2100] | 2000 [1600; 2500] |
| 90^th^ to 100^th^ | 2100 [1700; 2700] | 1700 [1200; 2400] | 2300 [1800; 3000] |
| **The Philippines** (TB treatment costs ranging from $36 to $618 and  costs of Xpert Ultra from $12 to $26) | | | |
| **Treatment and non-cartridge Xpert Ultra cost percentile** | **Cost per DALY averted: Universal Xpert** | **Cost per DALY averted: CRP testing** | **Cost per DALY averted: Hypothetical Screening test** |
| 0 to 10^th^ | 420 [300; 540] | 360 [260; 480] | 430 [270; 560] |
| 10^th^ to 20^th^ | 510 [420; 620] | 430 [340; 550] | 540 [440; 660] |
| 20^th^ to 30^th^ | 570 [470; 690] | 470 [370; 600] | 610 [500; 740] |
| 30^th^ to 40^th^ | 620 [510; 750] | 510 [400; 650] | 670 [560; 820] |
| 40^th^ to 50^th^ | 660 [550; 800] | 540 [430; 690] | 730 [600; 900] |
| 50^th^ to 60^th^ | 710 [590; 870] | 580 [460; 740] | 790 [660; 980] |
| 60^th^ to 70^th^ | 760 [630; 930] | 620 [480; 790] | 860 [700; 1100] |
| 70^th^ to 80^th^ | 810 [680; 1000] | 650 [520; 850] | 920 [760; 1100] |
| 80^th^ to 90^th^ | 880 [730; 1100] | 690 [550; 900] | 1000 [840; 1200] |
| 90^th^ to 100^th^ | 1000 [810; 1300] | 790 [610; 1100] | 1200 [920; 1500] |
| **South Africa** (TB treatment costs ranging from $111 to $1887 and  costs of Xpert Ultra from $15 to $55) | | | |
| **Treatment and non-cartridge Xpert Ultra cost percentile** | **Cost per DALY averted: Universal Xpert** | **Cost per DALY averted: CRP testing** | **Cost per DALY averted: Hypothetical Screening test** |
| 0 to 10^th^ | 880 [570; 1100] | 740 [480; 1000] | 950 [600; 1300] |
| 10^th^ to 20^th^ | 1100 [870; 1400] | 920 [690; 1300] | 1200 [970; 1600] |
| 20^th^ to 30^th^ | 1200 [1000; 1600] | 1100 [810; 1400] | 1400 [1100; 1800] |
| 30^th^ to 40^th^ | 1400 [1100; 1700] | 1200 [880; 1600] | 1600 [1300; 2000] |
| 40^th^ to 50^th^ | 1500 [1200; 1800] | 1200 [920; 1700] | 1700 [1400; 2100] |
| 50^th^ to 60^th^ | 1600 [1300; 2000] | 1300 [1000; 1800] | 1800 [1500; 2300] |
| 60^th^ to 70^th^ | 1700 [1400; 2200] | 1400 [1100; 2000] | 2000 [1600; 2600] |
| 70^th^ to 80^th^ | 1800 [1500; 2300] | 1500 [1200; 2100] | 2100 [1700; 2700] |
| 80^th^ to 90^th^ | 2000 [1600; 2500] | 1700 [1300; 2300] | 2300 [1800; 2900] |
| 90^th^ to 100^th^ | 2300 [1800; 3000] | 1900 [1400; 2600] | 2700 [2100; 3500] |

| **Uganda** (TB treatment costs ranging from $47 to $790 and  costs of Xpert Ultra from $13 to $34) | | | |
| --- | --- | --- | --- |
| **Treatment and non-cartridge Xpert Ultra cost percentile** | **Cost per DALY averted: Universal Xpert** | **Cost per DALY averted: CRP testing** | **Cost per DALY averted: Hypothetical Screening test** |
| 0 to 10^th^ | 1300 [940; 1700] | 1100 [760; 1500] | 1300 [860; 1700] |
| 10^th^ to 20^th^ | 1500 [1200; 1900] | 1200 [940; 1700] | 1600 [1300; 2100] |
| 20^th^ to 30^th^ | 1700 [1300; 2100] | 1400 [980; 1900] | 1800 [1400; 2300] |
| 30^th^ to 40^th^ | 1800 [1500; 2300] | 1400 [1100; 2000] | 2000 [1600; 2600] |
| 40^th^ to 50^th^ | 2000 [1600; 2500] | 1500 [1100; 2200] | 2200 [1700; 2800] |
| 50^th^ to 60^th^ | 2100 [1700; 2700] | 1600 [1200; 2300] | 2400 [1900; 3000] |
| 60^th^ to 70^th^ | 2200 [1800; 2800] | 1700 [1200; 2300] | 2500 [2000; 3200] |
| 70^th^ to 80^th^ | 2400 [1900; 3000] | 1800 [1300; 2500] | 2700 [2200; 3500] |
| 80^th^ to 90^th^ | 2600 [2000; 3200] | 1900 [1400; 2600] | 3000 [2400; 3800] |
| 90^th^ to 100^th^ | 2800 [2200; 3700] | 2100 [1500; 3000] | 3400 [2600; 4400] |
| **Vietnam** (TB treatment costs ranging from $49 to $834 and  costs of Xpert Ultra from $13 to $37) | | | |
| **Treatment and non-cartridge Xpert Ultra cost percentile** | **Cost per DALY averted: Universal Xpert** | **Cost per DALY averted: CRP testing** | **Cost per DALY averted: Hypothetical Screening test** |
| 0 to 10^th^ | 1000 [730; 1400] | 860 [600; 1200] | 1000 [650; 1400] |
| 10^th^ to 20^th^ | 1200 [950; 1600] | 1000 [750; 1400] | 1300 [990; 1700] |
| 20^th^ to 30^th^ | 1300 [1100; 1800] | 1100 [800; 1500] | 1400 [1100; 1900] |
| 30^th^ to 40^th^ | 1400 [1100; 1900] | 1200 [870; 1600] | 1600 [1200; 2000] |
| 40^th^ to 50^th^ | 1500 [1200; 2000] | 1200 [900; 1700] | 1700 [1300; 2200] |
| 50^th^ to 60^th^ | 1700 [1300; 2100] | 1300 [940; 1800] | 1800 [1400; 2400] |
| 60^th^ to 70^th^ | 1800 [1400; 2300] | 1400 [1000; 1900] | 2000 [1600; 2600] |
| 70^th^ to 80^th^ | 1900 [1500; 2400] | 1400 [1100; 2000] | 2100 [1700; 2800] |
| 80^th^ to 90^th^ | 2000 [1600; 2700] | 1600 [1100; 2200] | 2400 [1800; 3100] |
| 90^th^ to 100^th^ | 2300 [1800; 3000] | 1700 [1300; 2400] | 2700 [2100; 3600] |

# Table S7. Incremental cost-effectiveness of each case-finding strategy assuming all parameters to follow a uniform distribution.

| Scenario | Country | Willingness to pay (2023 USD per DALY averted) | Incremental cost-effectiveness ratio (2023 USD per DALY averted) | |
| --- | --- | --- | --- | --- |
|  |  |  | Vs. Xpert Ultra for all [2.5th; 97.5] | Vs. No case-finding [2.5th; 97.5] |
| CRP –  Scenario 2 | India | 560 | 1800 [1200, 3100] | 1400 [870, 2300] |
|  | Philippines | 1061 | 830 [550, 1400] | 580 [380, 920] |
|  | South Africa | 3725 | 1800 [1200, 2900] | 1400 [830, 2300] |
|  | Uganda | 192 | 2100 [1200, 3900] | 1400 [800, 2400] |
|  | Vietnam | 712 | 2500 [1500, 4500] | 1700 [990, 2900] |
|  | **Mean** | **1250** | **1800 [1100, 3100]** | **1300 [770, 2200]** |
| Hypothetical Screening test –  Scenario 3a | India | 560 | 1400 [770, 2900] | 1600 [1100, 2400] |
|  | Philippines | 1061 | 330 [-87, 600] | 770 [540, 1100] |
|  | South Africa | 3725 | 440 [-930, 1000] | 1800 [1200, 2700] |
|  | Uganda | 192 | 710 [-486, 1600] | 1900 [1200, 3100] |
|  | Vietnam | 712 | 860 [-435, 1800] | 2300 [1500, 3600] |
|  | **Mean** | **1250** | **740 [-234, 1600]** | **1700 [1100, 2600]** |
| Xpert Ultra for all –  Scenario 4 | India | 560 | -- | 1600 [1100, 2200] |
|  | Philippines | 1061 | -- | 690 [490, 990] |
|  | South Africa | 3725 | -- | 1600 [1100, 2300] |
|  | Uganda | 192 | -- | 1700 [1100, 2700] |
|  | Vietnam | 712 | -- | 2000 [1400, 3100] |
|  | **Mean** | **1250** | **--** | **1500 [1000, 2300]** |

# Figure S1. One-way sensitivity analysis on the incremental cost-effectiveness of CRP testing versus no screening for tuberculosis.

Shown is the incremental cost-effectiveness ratio (ICER, x-axis) of screening with the CRP test for TB in each country, relative to no screening, under one-way variation of key model parameters (range given below each parameter, baseline values are presented in the main manuscript text, Table 1). Parameters for which variation did not change ICER estimates by more than ±10% are not shown. The numbers to the left and right of each bar show the high and low values of the ICER (rounded to two significant digits) when varying the respective parameter (blue bars = upper bound of the parameter’s range, red bars = lower bound of the parameter’s range [there is no lower bound for loss to follow up, as the loss to follow up was already at 0% in the baseline model]). The black vertical line specifies the ICER when all parameters are held at their baseline values. The dashed vertical line represents each country’s willingness-to-pay per additional DALY averted. This analysis for India is shown as Figure 3 in the main text.

a) The Philippines

**
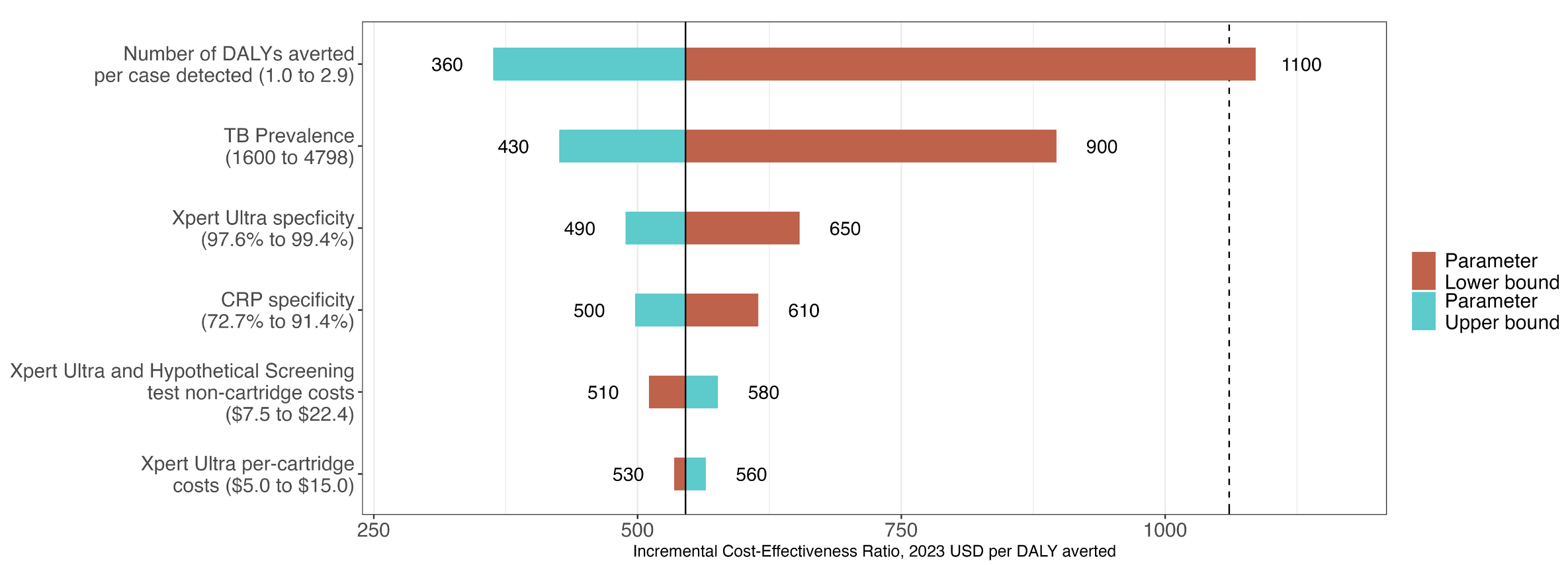
**

b) South Africa


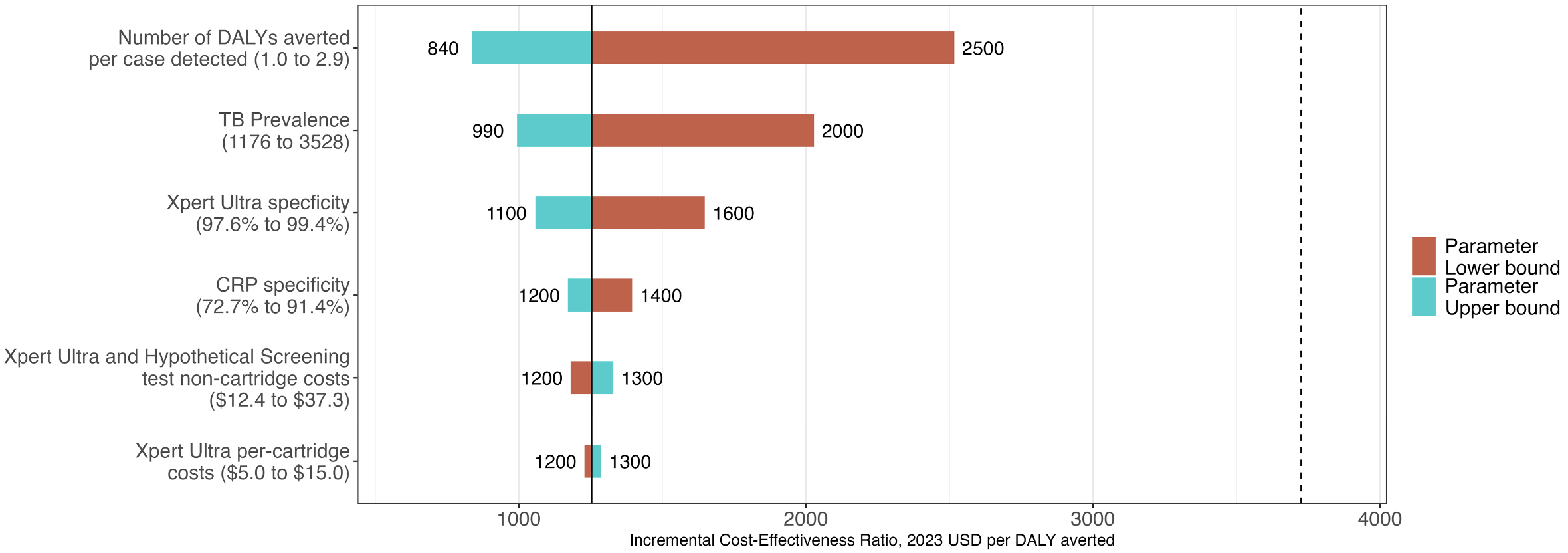


c) Uganda


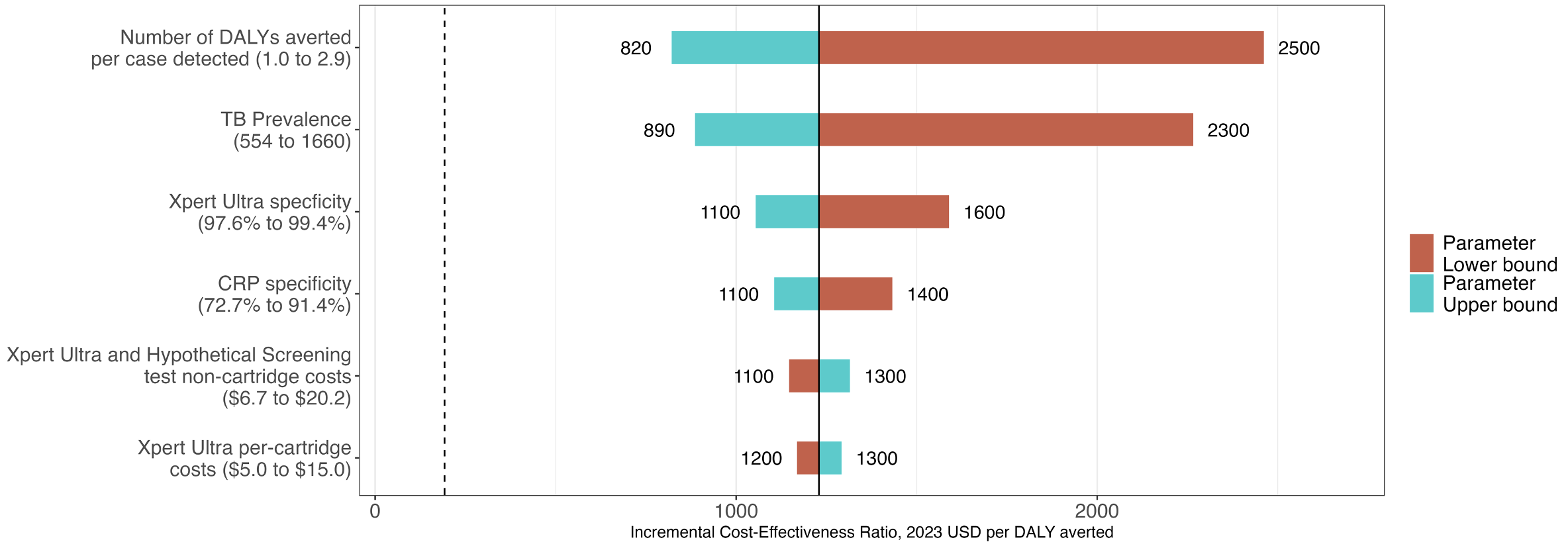


d) Vietnam


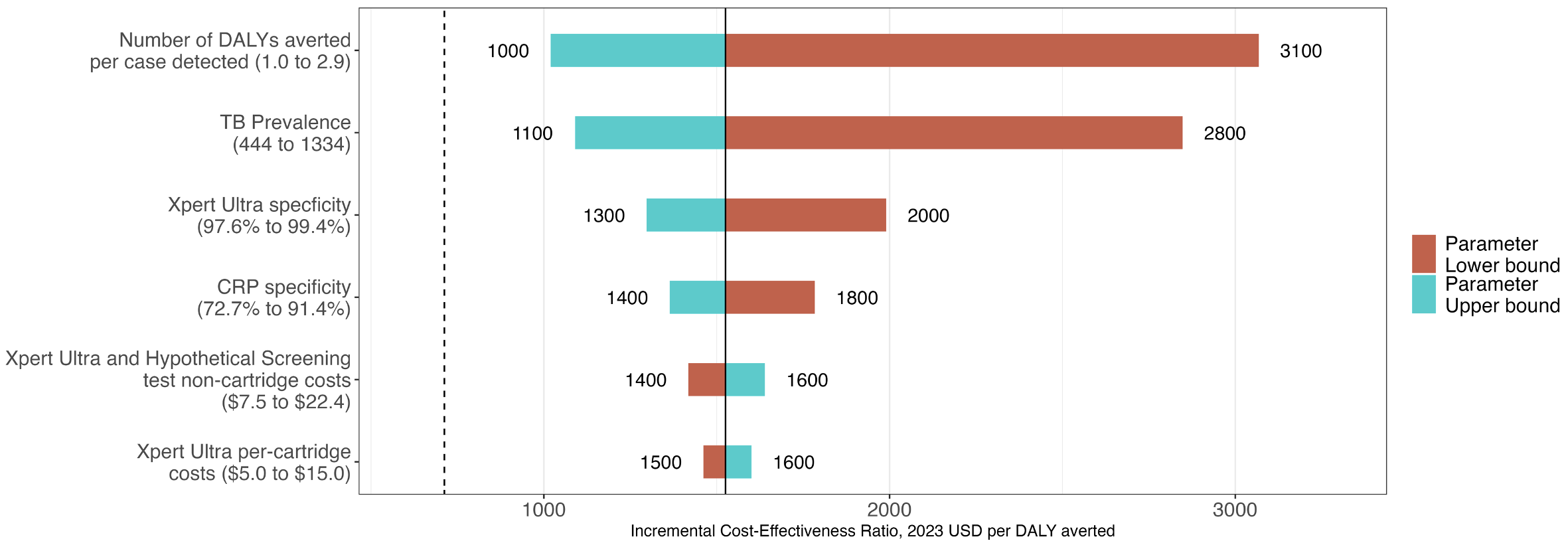


# Figure S2. One-way sensitivity analysis on the incremental cost-effectiveness of the Hypothetical Screening test versus no screening for tuberculosis.

*Caption:* Shown is the incremental cost-effectiveness ratio (ICER, x-axis) of screening with the Hypothetical Screening test for TB in the respective country, relative to no screening, under one-way variation of key model parameters (range given below each parameter, baseline values are presented in the main manuscript, Table 1). Parameters for which variation did not change ICER estimates by more than ±10% are not shown. The numbers to the left and right of each bar show the high and low values of the ICER (rounded to two significant digits) when varying the respective parameter (blue bars = upper bound of the parameter’s range, red bars = lower bound of the parameter’s range [there is no lower bound for loss to follow up, as the loss to follow up was already at 0% in the baseline model]). The dashed vertical line represents each country’s willingness-to-pay per additional DALY averted.

a) India


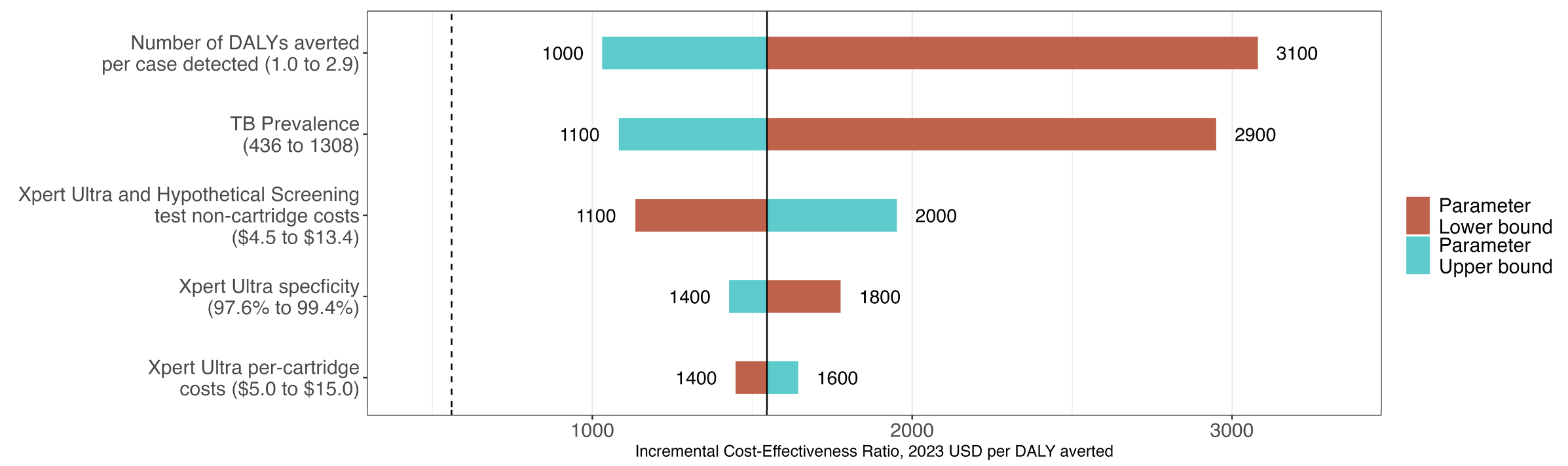


b) The Philippines


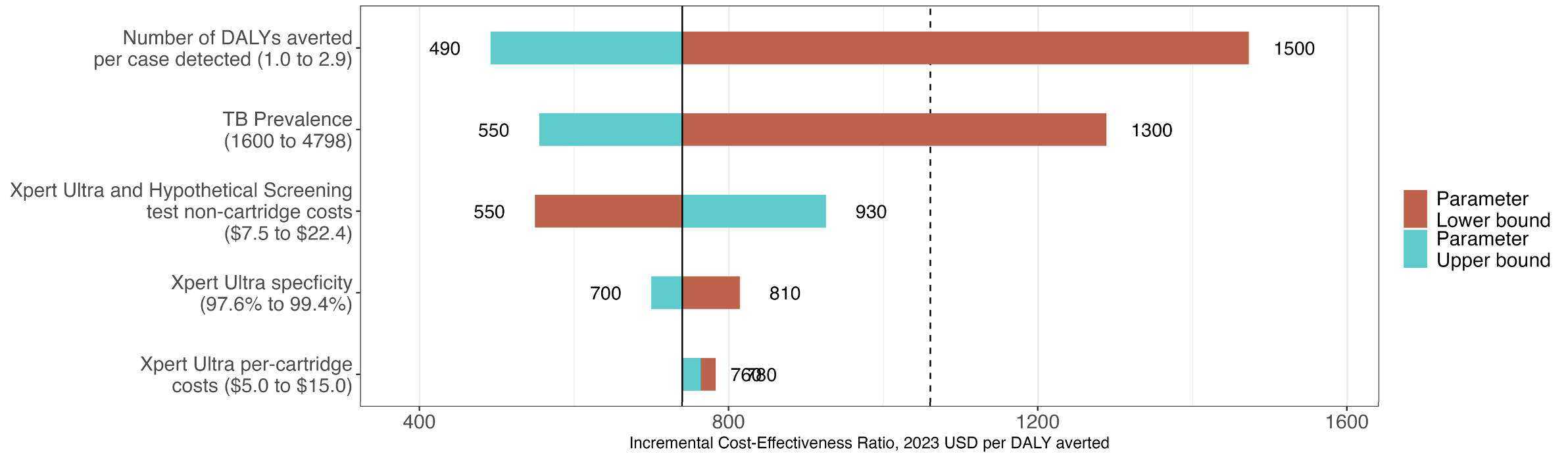


c) South Africa


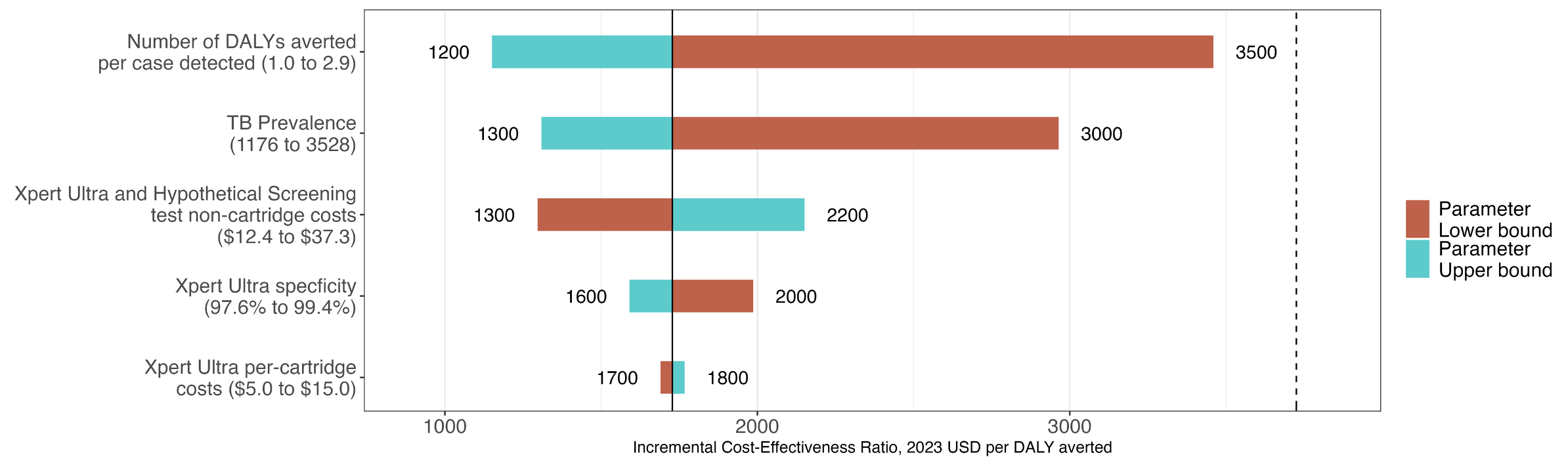


d) Uganda


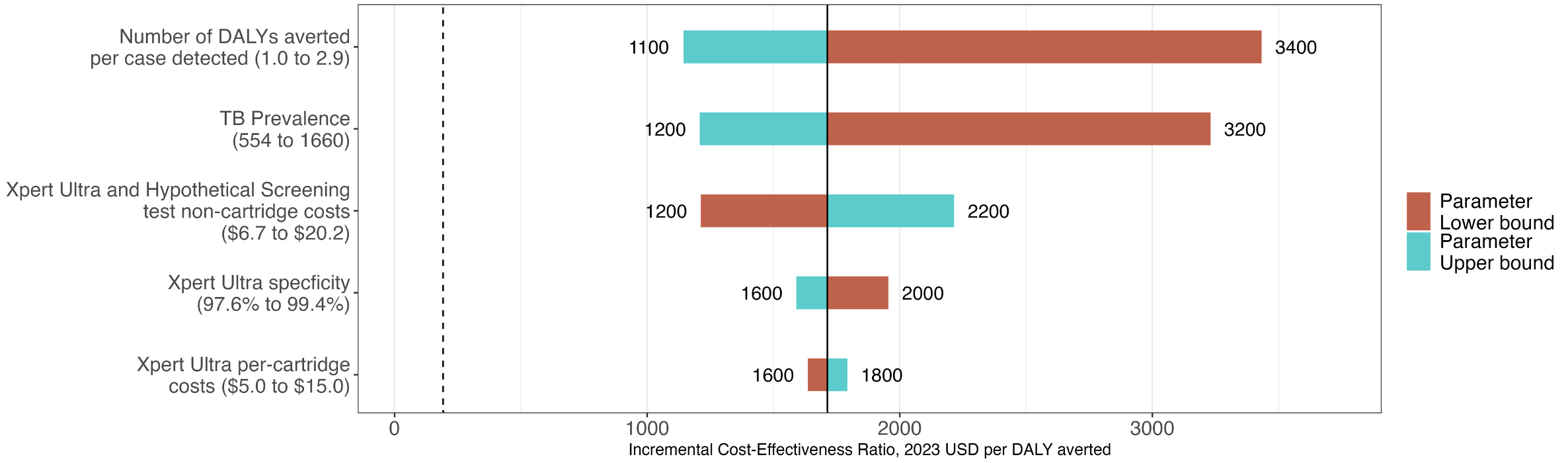


e) Vietnam


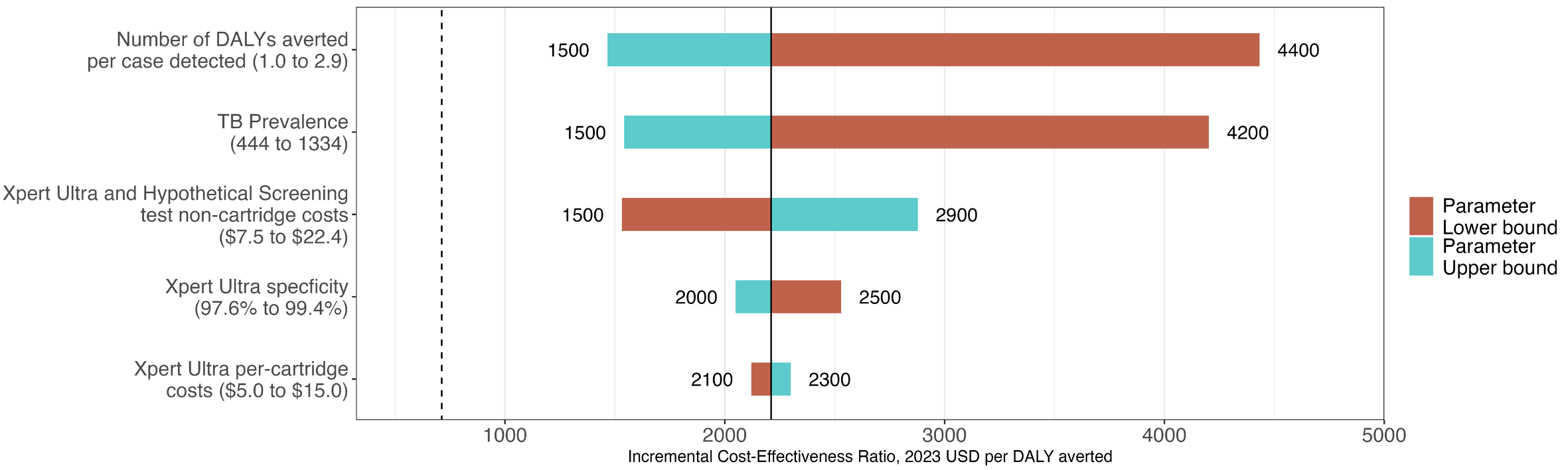


# Figure S3. Efficiency frontiers for different community-based screening approaches for tuberculosis in Uganda including screening with chest X-ray and symptom screen.

The present figure shows total costs in 2023 United States dollars (USD, y-axis, in millions) of each screening strategy plotted against the number of disability adjusted life years (DALYs) averted (x-axis, in thousands), per 100,000 people screened in a high-prevalence (four-times the national prevalence), Uganda-like setting. Each triangle, square, and circle represents the costs per DALY averted of the respective testing strategies as listed in legend to the right of the figure, with the costs per DALY averted compared to the next cheaper strategy written above each symbol for those strategies on the efficiency frontier (further details in the caption of Figure 1 in the main manuscript, which is also replicated here as panel A for convenience of comparison). In addition to the strategies included in the main manuscript (universal Xpert Ultra, Hypothetical Screening test, CRP test, no case-finding), panel B of present figure shows the costs per DALY averted if active case-finding were conducted through screening for cough >2 weeks (dark blue triangle; 42% [36% to 48%] sensitivity for Xpert Ultra positive TB, 94% [92% to 96%] specificity, costs of $2.21 [$1.66 to $2.76] per test all-inclusive) or screening using chest-X-ray (dark green triangle; 85% [77% to 90%] sensitivity for Xpert Ultra positive TB, 96% [93% to 97%] specificity, costs of $10.27 [$7.70 to $12.84] per test all-inclusive) [1-5].


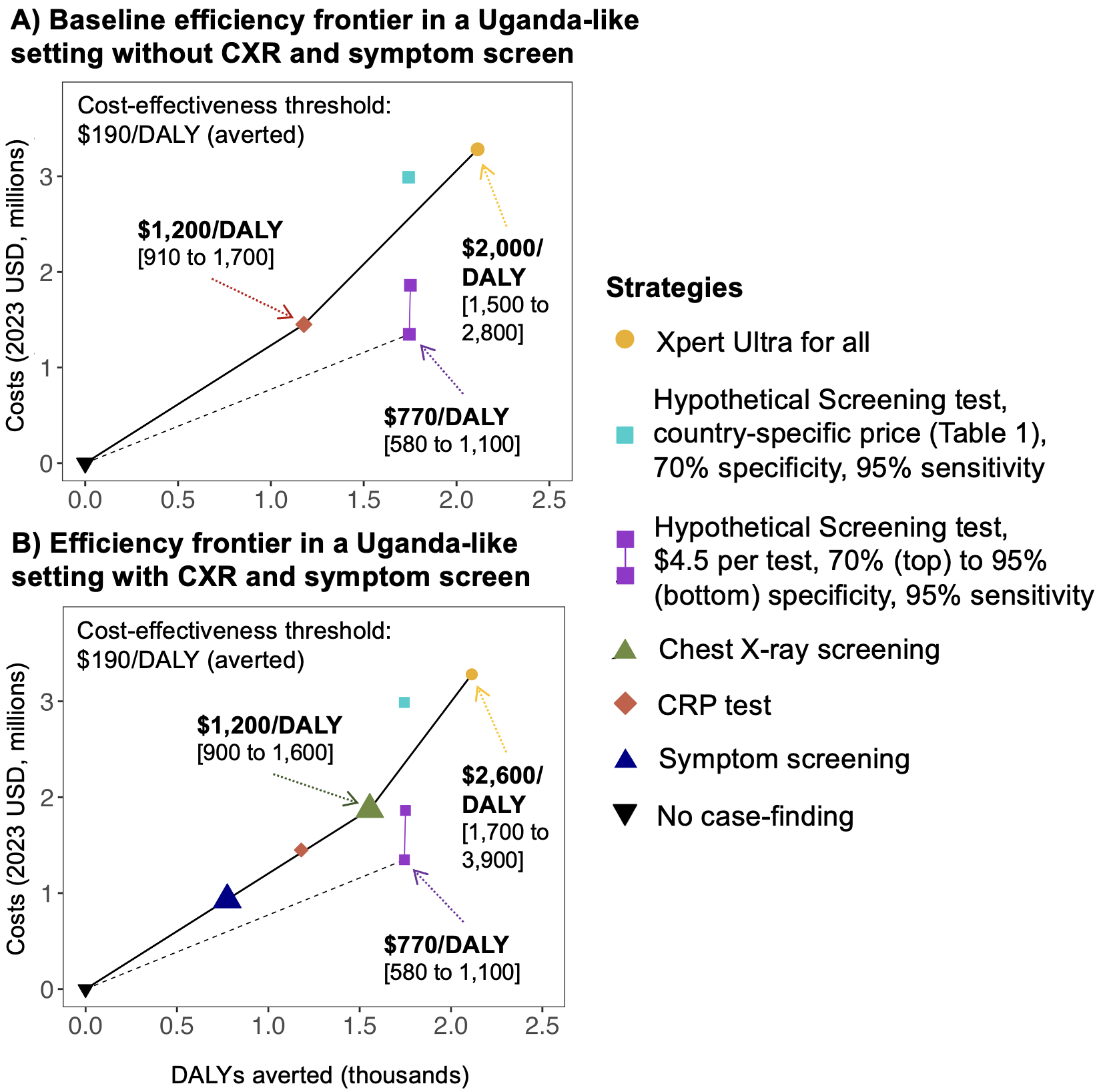


*Sources*

[1] *World Health Organization. WHO consolidated guidelines on tuberculosis: module 2: screening: systematic screening for tuberculosis disease, 2021 update. Geneva; 2021.*

[2] *Machekera SM, Wilkinson E, Hinderaker SG, Mabhala M, Zishiri C, Ncube RT, et al. A comparison of the yield and relative cost of active tuberculosis case-finding algorithms in Zimbabwe. Public Health Action. 2019;9(2):63-8.*

[3] *Global Drug Facility. Diagnostics, medical devices & other health products catalog. March 2023. Available online:* [*https://www.stoptb.org/sites/default/files/gdf_diagnostics_medical_devices_other_health_products_catalog_0.pdf*](https://www.stoptb.org/sites/default/files/gdf_diagnostics_medical_devices_other_health_products_catalog_0.pdf)

[4] *Stop TB Partnership. Artificial intelligence-powered computer-aided detection (CAD) software. January 2022.* [*https://www.stoptb.org/introducing-new-tools-project/artificial-intelligence-powered-computer-aided-detection-cad-software*](https://www.stoptb.org/introducing-new-tools-project/artificial-intelligence-powered-computer-aided-detection-cad-software)

[5] *Baik Y, Nakasolya O, Isooba D, Mukiibi J, Kitonsa PJ, Erisa KC, et al. Cost to perform door-to-door universal sputum screening for TB in a high-burden community. Int J Tuberc Lung Dis. 2023;27(3):195-201.*

# Figure S4. Scenario analysis: efficiency frontiers for CRP screening in an HIV-positive clinic-based population.

The present figure shows total costs in 2023 United States dollars (USD, y-axis, in millions) of each screening strategy plotted against the number of disability adjusted life years (DALYs) averted (x-axis, in thousands), per 100,000 people screened in a high-prevalence (four-times the national prevalence), Uganda-like setting. Each triangle, square, and circle represents the costs per DALY averted of the respective testing strategies as listed in legend to the right of the figure, with the costs per DALY averted compared to the next cheaper strategy written above each symbol for those strategies on the efficiency frontier (further details in the caption of Figure 1 in the main manuscript). In contrast to the 65% sensitivity for Xpert Ultra positive TB and 84% specificity assumed for CRP in the main manuscript, this figure presents the projected costs per DALY averted when considering CRP to be 96% sensitive for Xpert Ultra positive TB and 12% specific (as it might be found testing an HIV-positive clinic-based population) [1].


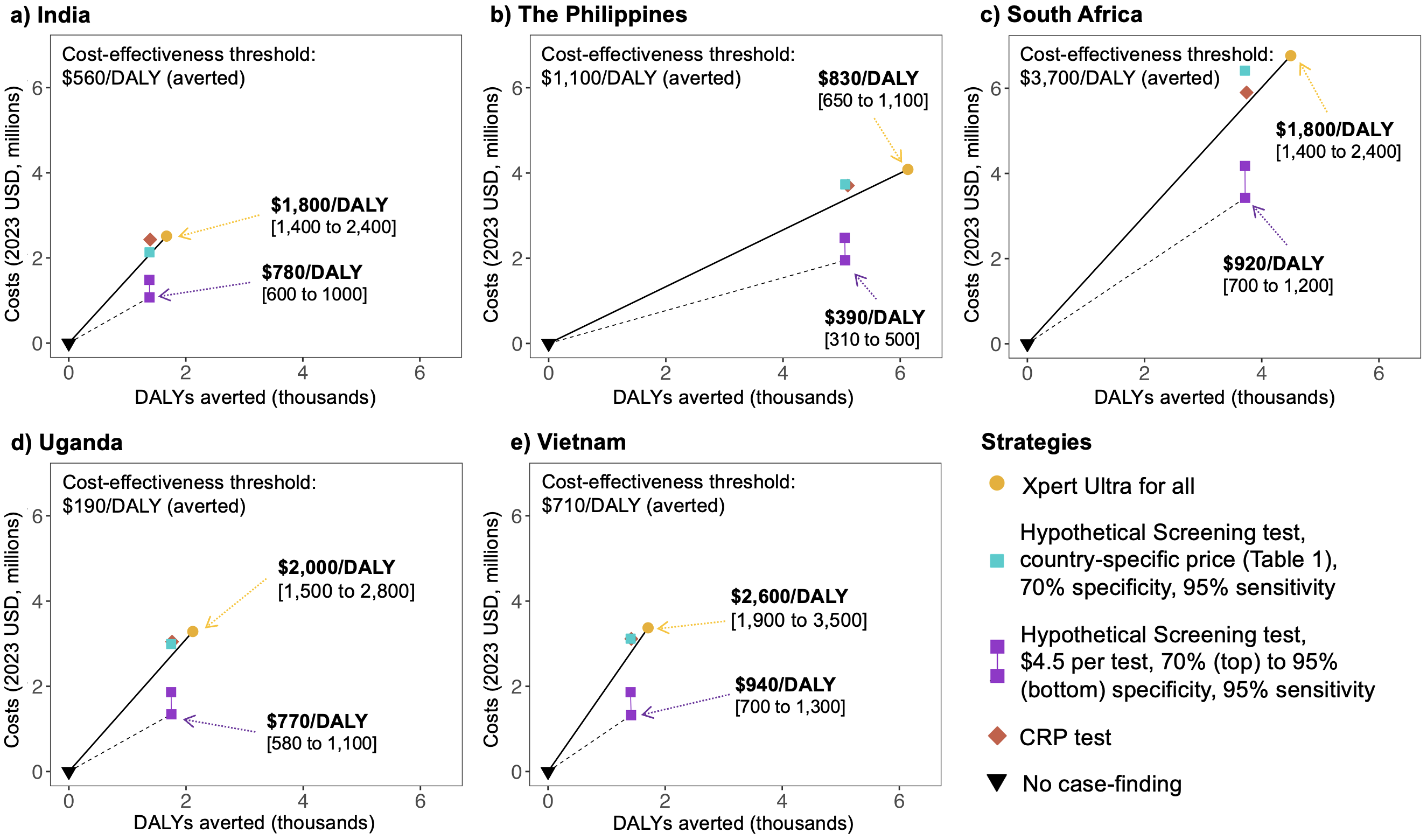


Sources

[1] *Dhana A, Hamada Y, Kengne AP, Kerkhoff AD, Rangaka MX, Kredo T, et al. Tuberculosis screening among HIV-positive inpatients: a systematic review and individual participant data meta-analysis. Lancet HIV. 2022;9(4):e233-e41.*

# Figure S5. Scenario analysis: efficiency frontiers for different community-based screening approaches for tuberculosis in South Africa using lowest Xpert Ultra and treatment cost estimates.

The present figure shows total costs in 2023 United States dollars (USD, y-axis, in millions) of each screening strategy plotted against the number of disability adjusted life years (DALYs) averted (x-axis, in thousands), per 100,000 people screened in a high-prevalence (four-times the national prevalence), South Africa-like setting. Each triangle, square, and circle represents the costs per DALY averted of the respective testing strategies as listed in the legend to the right of the figure, with the costs per DALYs averted compared to the next cheaper strategy written above each symbol for those strategies on the efficiency frontier (further details in the caption of Figure 1 in the main manuscript). Panel A shows the same results as Figure 1 panel C in the main manuscript, i.e., the results of the baseline analysis assuming TB treatment per-person costs of $999 (95% uncertainty range [UR]: $749 to $1,249) [1] and Xpert Ultra non-cartridge per-test costs of $24.87 (95% UR: $22.58 to $27.15) [2]. As a scenario analysis accounting for different cost estimates found in studies specific to South Africa, panel B assumes per-person costs of TB treatment of $111 (95% uncertainty range [UR]: $83 to $139) [3] and panel C considers non-cartridge per-test costs of Xpert Ultra of $4.97 (95% UR: $4.52 to $5.43) [4]. Panel D shows results of combining the low treatment costs shown in panel B and the low Xpert-Ultra non-cartridge cost estimates shown in panel C.


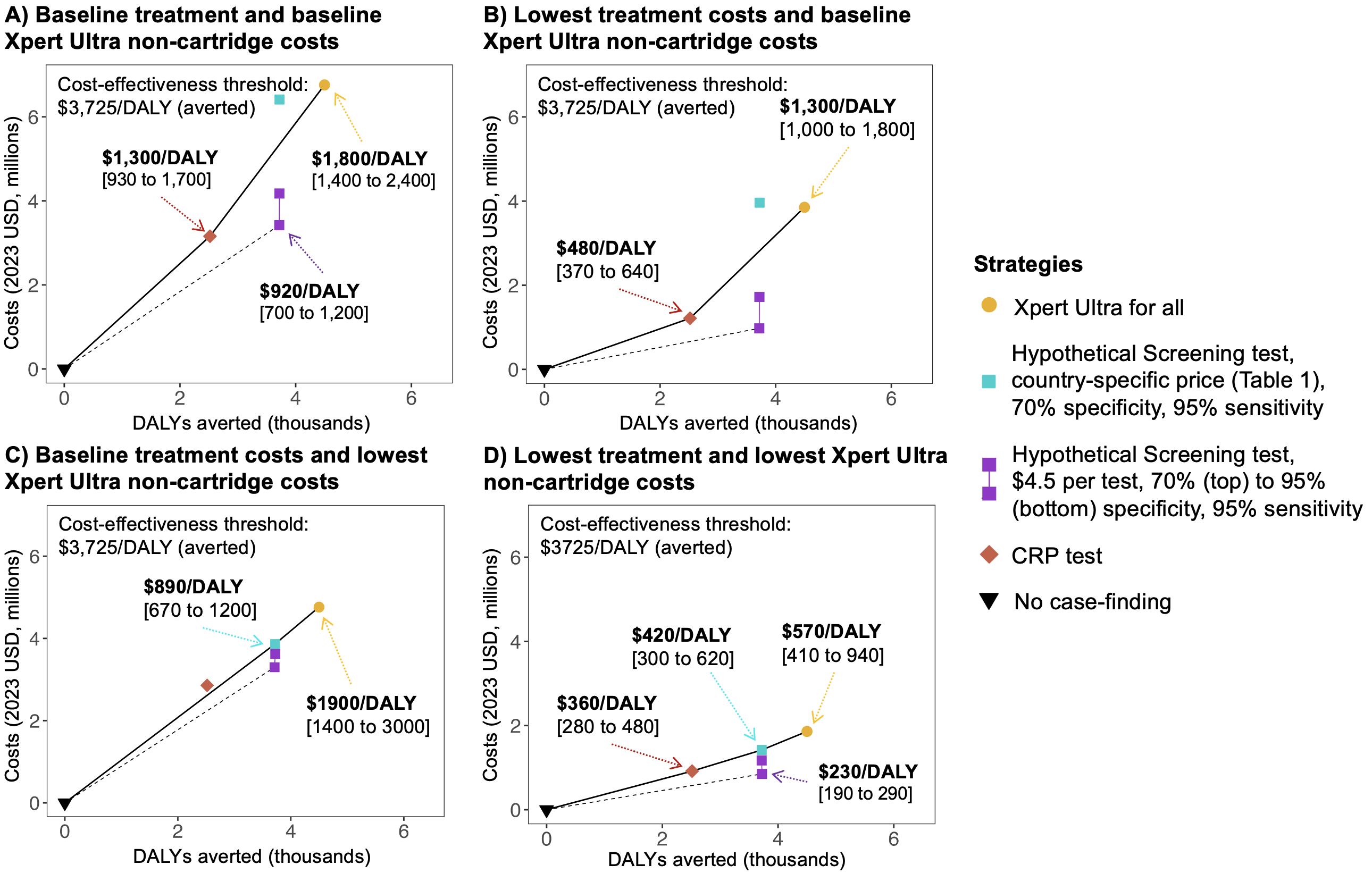


Sources

[1] *World Health Organization. Global tuberculosis report 2020. Geneva (Switzerland); 2020.*

[2] *Pooran A, Theron G, Zijenah L, Chanda D, Clowes P, Mwenge L, et al. Point of care Xpert MTB/RIF versus smear microscopy for tuberculosis diagnosis in southern African primary care clinics: a multicentre economic evaluation. Lancet Glob Health. 2019;7(6):e798-e807.*

[3] *Kgowedi S, Girdwood S, Govender K, Lekodeba N, Meyer-Rath G, Miot J, Long L. Cost and outcomes of drug susceptible TB treatment at primary healthcare clinics. Johannesburg: HE2RO Policy Brief Number 33, Health Economics and Epidemiology Research Office, 2020. Available online:* [*https://www.heroza.org/wp-content/uploads/2020/10/Policy-Brief-37-Tuberculosis.pdf*](https://www.heroza.org/wp-content/uploads/2020/10/Policy-Brief-37-Tuberculosis.pdf)

[4] *Cassim N, Coetzee LM, Makuraj AL, Stevens WS, Glencross DK. Establishing the cost of Xpert MTB/RIF mobile testing in high-burden peri-mining communities in South Africa. Afr J Lab Med. 2021;10(1):1229*
